# HPV DeepSeq: An Ultra-Fast Method of NGS Data Analysis and Visualization Using Automated Workflows and a Customized Papillomavirus Database in CLC Genomics Workbench

**DOI:** 10.3390/pathogens10081026

**Published:** 2021-08-13

**Authors:** Jane Shen-Gunther, Qingqing Xia, Hong Cai, Yufeng Wang

**Affiliations:** 1 Gynecologic Oncology & Clinical Investigation, Department of Clinical Investigation, Brooke Army Medical Center, Fort Sam Houston, San Antonio, TX 78234, USA; 2 Department of Clinical Investigation, Brooke Army Medical Center, Fort Sam Houston, San Antonio, TX 78234, USA; qingqing.xia.civ@mail.mil; 3 Department of Biology, University of Texas at San Antonio, San Antonio, TX 78249, USA; hong.cai@utsa.edu; 4 South Texas Center for Emerging Infectious Diseases, University of Texas at San Antonio, San Antonio, TX 78249, USA

**Keywords:** bioinformatics, cervical cancer, deep sequencing, human papillomavirus, HPV genotyping, metagenome, next generation sequencing, taxonomic classification, virome

## Abstract

Next-generation sequencing (NGS) has actualized the human papillomavirus (HPV) virome profiling for in-depth investigation of viral evolution and pathogenesis. However, viral computational analysis remains a bottleneck due to semantic discrepancies between computational tools and curated reference genomes. To address this, we developed and tested automated workflows for HPV taxonomic profiling and visualization using a customized papillomavirus database in the CLC Microbial Genomics Module. HPV genomes from Papilloma Virus Episteme were customized and incorporated into CLC “ready-to-use” workflows for stepwise data processing to include: (1) Taxonomic Analysis, (2) Estimate Alpha/Beta Diversities, and (3) Map Reads to Reference. Low-grade (*n* = 95) and high-grade (*n* = 60) Pap smears were tested with ensuing collective runtimes: Taxonomic Analysis (36 min); Alpha/Beta Diversities (5 s); Map Reads (45 min). Tabular output conversion to visualizations entailed 1–2 keystrokes. Biodiversity analysis between low- (LSIL) and high-grade squamous intraepithelial lesions (HSIL) revealed loss of species richness and gain of dominance by HPV-16 in HSIL. Integrating clinically relevant, taxonomized HPV reference genomes within automated workflows proved to be an ultra-fast method of virome profiling. The entire process named “HPV DeepSeq” provides a simple, accurate and practical means of NGS data analysis for a broad range of applications in viral research.

## 1. Introduction

Hippocrates was the first to describe cervical cancer and its destructive nature around 400 BCE [1]. Two thousand years elapsed before zur Hausen and his “papillomavirus crew” made the breakthrough discovery of identifying human papillomavirus (HPV)-16 in cervical, vulvar, and penile cancers in 1983 [1,2,3]. Since then, the causal role of carcinogenic HPV in anogenital, oropharyngeal, and dermatological cancers (in patients with epidermodysplasia verruciformis) has been established and classified by the International Agency for Research on Cancer (IARC) [4,5]. The etiological role of HPV in breast and esophageal cancers has been postulated for several decades but remains controversial due to conflicting findings [6,7]. Nonetheless, HPV is the second most prevalent primary infectious cause of cancer worldwide. The annual global burden of 570,000 new cervical and 120,000 other anogenital and oropharyngeal cancer cases have been attributed to HPV [8,9].

The papillomavirus (PV) is a small 8000 base pair (bp) double-stranded, circular DNA virus that co-evolved with an ancestral host over 400 million years [10]. The PV genome backbone acquired oncogenes E6 and E7 and later E5 approximately 184 and 55 million years ago, respectively [10]. The genetic differences in these oncogenes conferred disparate phenotypes and oncogenic potential [10,11]. Recent phylogenetic analysis also suggested that anatomical site predilection and tissue tropism by distinct HPV genotypes may be a result of viral niche adaptation to host ecosystems [12]. Site-specific genotypes and virome composition may be further shaped by the host’s immune response [13]. Therefore, anatomical virome characterization is crucial to our understanding of niche-specific, virus-host adaptive evolution foundational to pathogenesis.

Recent advancements in next-generation sequencing (NGS) have actualized HPV virome profiling [14]. Since the commercialization of high-throughput sequencing (HTS) instruments in 2005, the variety and choices of sequencing technologies, chemistries, platforms, capacities, and kits have expanded exponentially [15,16]. The wet lab portion of genomics research has become more streamlined, simpler, and accessible to the researcher. However, bioinformatics analysis remains a bottleneck [17,18]. First, the enormous amounts of complex data generated from each sample is non-trivial. This is compounded by disparate, open-source tools which are often command-line based and require advanced computational or coding skills [17,18]. Second, viral taxonomies based on the International Committee on Taxonomy of Viruses (ICTV) are subject to new revisions. In 2019, the ICTV taxonomic structure was revised from a 5-rank (1991–2017) to a 15-rank structure which imposes retooling of existing software and restructuring of reference database(s) for viral metagenomic analysis [19]. Furthermore, the 15-rank format is inconsistent with that of open-source Quantitative Insights into Microbial Ecology (QIIME) popularly used for bacterial and fungal taxonomic analysis [20]. To circumvent these two daunting barriers (i.e., taxonomic structure and software), we customized the curated Papilloma Virus Episteme (PaVE) database [21] for use in a user-friendly, GUI-based, commercial software for sequence analysis. We aimed to develop and test automated workflows for HPV taxonomic profiling and visualization within the CLC Microbial Genomics Module (MGM) plugged into the main Workbench (WB). A simple, rapid, and accurate means of NGS data analysis will ultimately propel HPV research and serve a broad range of applications from discovery to therapeutics.

## 2. Results

### 2.1. Taxonomic Classification and Visualization of HPV Metagenomes

This dataset included 155 cytology samples, classified as low-grade squamous intraepithelial lesions (LSIL) (*n* = 95) and high-grade squamous intraepithelial lesions (HSIL) (*n* = 60). The “Data quality control (QC) and taxonomic profiling” workflow computational runtime on this dataset was 23:33 and 12:50 min for the LSIL and HSIL samples, respectively (Table 1). The QC workflow generated the following outputs: (1) QC for sequencing reads (graphical report and supplementary report) and (2) Abundance table. Specifically, the graphical report summarized the total number of sequences and nucleotides in a sample, per-sequence analysis, per-base analysis, over-representation analyses, sequence duplication levels, and duplicated sequences. The QC supplementary report includes two additional columns (i.e., “coverage” and “abs”) for absolute numbers of sequences or bases for the per-sequence or per-base analyses. An in-depth explanation of the QC metrics is beyond the scope of this article. The reader is referred to the CLC MGM manual online for details.

The taxonomic profiling workflow generated individual abundance tables that display the names of the identified taxa, 7-level taxonomic nomenclature, coverage estimate, and abundance value (raw or relative number of reads found in the sample associated with the taxon). A merged abundance table inclusive of multiple samples also displays summary statistics (e.g., combined abundance of reads for the taxon across all samples, and the minimum, maximum, mean, median and standard deviation of the number of reads for the taxa across all samples) (Supplementary Table S1). Additional reports, such as, “Reads (mapping to database or host)” and “Reads (umapped)” may also be selected for output. However, this is performed at the cost of increased runtime.

**Figure 1:**
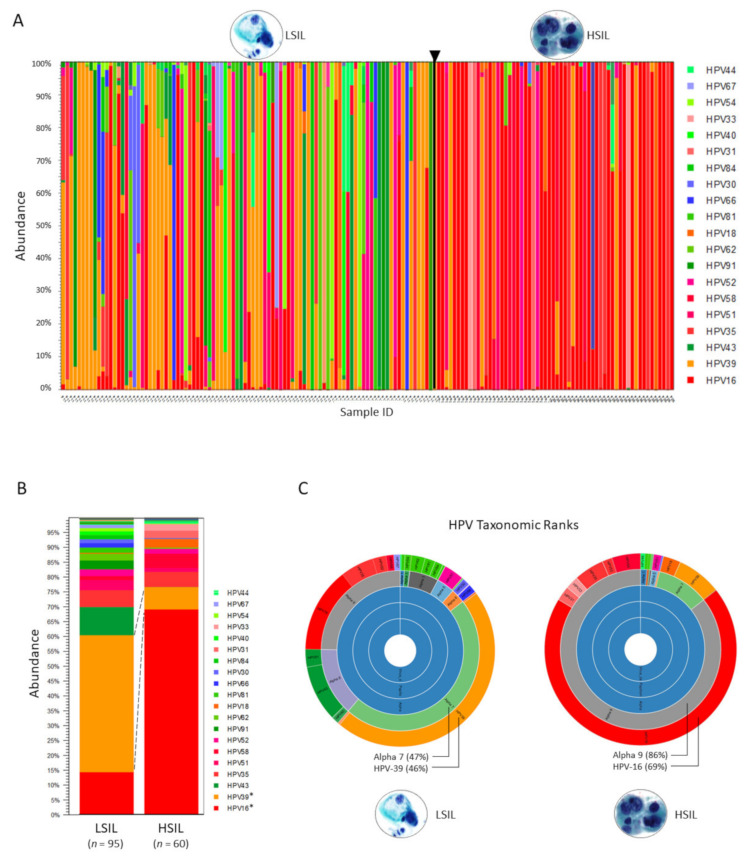
Taxonomic profiling results based on the HPV Reference Index. (**A**) Abundance of HPV genotypes found in individual LSIL and HSIL samples are shown as stacked bars. HPV type-specific carcinogenicity (carcinogenic, possibly carcinogenic, and not carcinogenic) are colorized in shades of red, blue, and green, respectively (legend). Deep sequencing of HPV E6/E7 amplicons derived from each LSIL (*n* = 95) or HSIL (*n* = 60) sample identified 32 unique HPV genotypes with the top 20 shown (legend) and quantitated their composition (%) based on abundance (*n*) of mapped reads to total mapped reads. (**B**) HPV genotype composition of samples grouped by cytological grade i.e., LSIL and HSIL. Visualization and comparison of grouped samples (stacked bars) revealed the dominant genotypes in LSIL and HSIL as HPV-39 (46%) and HPV-16 (69%), respectively with significant changes in proportional composition (Baggerley’s test, * *p*-value (Bonferroni) < 0.001). (**C**) Sunburst plots visualize hierarchical data outwardly from parent to child nodes. Here, sunburst plots reveal distinct differences in the HPV communities according to seven taxonomic ranks, specifically, the last two ranks (genus/species and genotypes) between LSIL and HSIL.

The abundance table may be visualized as a stacked bar chart or sunburst plot with 1-click (Figure 1A–C). Metadata added and used to aggregate groups of samples (i.e., cytological grades “LSIL” and “HSIL”) produced the 2-group stacked bar chart for comparison of HPV genotype composition (Figure 1B). The sunburst plots display hierarchical taxonomic ranks revealing distinct differences in the HPV communities between LSIL and HSIL groups (Figure 1C).

Both Sanger and NGS sequencing were used to detect HPV genotypes and sub-lineages within each sample. Sanger sequencing resolved the single dominant HPV genotype within each sample. Compared to Sanger sequencing, NGS achieved a higher resolution in detection of mixed genotypes (cut-off at six genotypes) and low-abundance genotypes (cut-off at ≥1% of total composition) (Supplementary Table S2). Comparing the dominant genotypes and sub-lineages (variants) derived from both sequencing methods, the inter-assay reliability was near-perfect (kappa = 0.94) (Table 2). Samples (9/155 samples, 5.81%) with discordant HPV genotyping results may be explained by: (1) low-quality Sanger sequence (BLAST “not available”) (*n* = 1), (2) low-quality Sanger sequence (BLAST max id < 90% and max bit score <531) (*n* = 2), (3) L1 and E6/E7 primer bias (*n* = 3), or (4) E6 sequencing primer bias (*n* = 3). The complete Sanger/BLAST table with 9 discordant results (bold) are shown in Supplementary Table S3.

By convention, sequences must possess ≥ 90% homology with the closest known type for HPV genotyping assignment [21]. Correlation analysis was performed to find the corresponding BLAST max bit score and E-value to BLAST 90% maximum identity. Two relationships emerged: (1) BLAST maximum bit score and log_10_ (E-value) were perfectly, linearly anti-correlated, and (2) BLAST max bit score and maximum identity (%) were curvilinearly correlated (Supplementary Figure S1). Taken together, BLAST maximum identity of ≥90% corresponded to a maximum bit score of ≥ 531 (equivalent to E-value ≤ log_10_ -150) which were used as the threshold for quality genotyping results. BLAST values less than this threshold were considered as “uncertain” HPV genotyping result.

### 2.2. Diversity Analysis and Visualization of LSIL/HSIL HPV Communities

The “Merge and Estimate Alpha and Beta Diversities” workflow runtime on this dataset was 5 s (Table 1). The diversity analyses workflow generated the following outputs: (1) alpha diversity rarefaction table and plots, and (2) beta diversity distance matrix and principal coordinate analysis (PCoA) plots.

**Figure 2:**
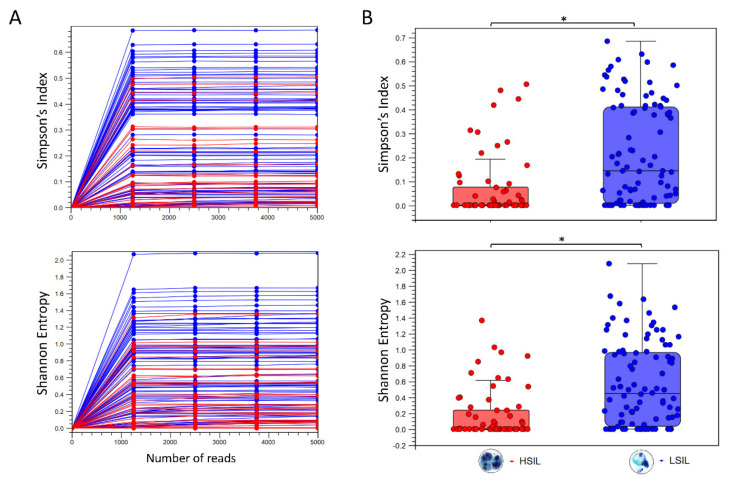
Diversity analysis of HPV genotypes in LSIL and HSIL samples. (**A**) Alpha diversity rarefaction curves estimate the indices for HPV genotypes in LSIL (*n* = 95) and HSIL (*n* = 60) samples. (**B**) Summary statistics are shown as boxplots after categorical grouping. A total of 29 unique genotypes were found in LSIL versus 22 genotypes for HSIL. Species richness measured by Simpson’s index showed a reduction from LSIL (median = 0.146) to HSIL (median = 0.062) samples (Mann-Whitney test, * *p* < 0.001). The respective Shannon indices for LSIL (median = 0.454) and HSIL (median = 0.215) were indicative of reduced diversity with disease progression (Mann-Whitney test, * *p* < 0.001).

The alpha diversity metric is calculated by sub-sampling the abundances at different depths (number of reads) in each sample. The rarefaction analysis parameters define the granularity of the alpha diversity curve as presented in Figure 2. Abundance table metadata used for aggregating groups of samples (i.e., “LSIL” and “HSIL”) produced the box plot with auto-calculated Mann-Whitney U statistical results in Figure 2.

**Figure 3:**
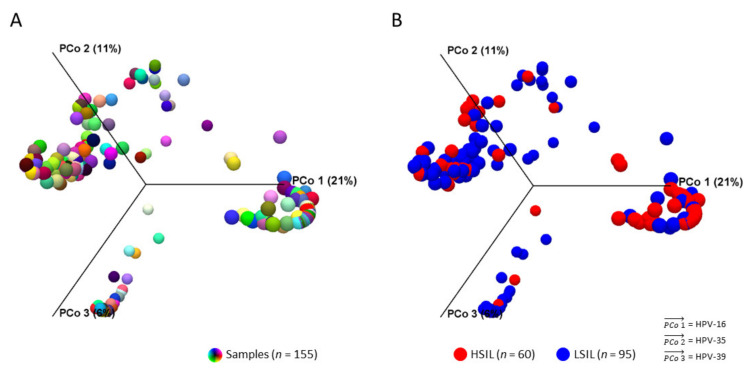
HPV community structures between LSIL and HSIL. 3D-Principal Coordinate Analysis (PCoA) plots of samples (*n* = 155) in distinct colors before (**A**) and after (**B**) grouping by cytological category. After grouping by LSIL and HSIL, HPV-16, -35, -39 were identified as the three most influential genotypes (vectors) in both HPV communities. Dissimilarity between the two HPV communities was HPV-16 (PCoA 1) being the most influential genotype in HSIL versus HPV-39 for LSIL (PCoA 3). β-diversity was measured by Bray-Curtis index (PERMANOVA, *p* < 0.05).

The workflow output for beta diversity analysis was a Bray-Curtis distance matrix between samples and PCoA plots in 2D or 3D. The 3D PCoA plot visually displayed the dissimilarities in HPV composition between all samples (Figure 3A). After grouping HSIL and LSIL samples, the dissimilar HPV communities and the most influential genotypes were visually apparent (Figure 3B).

### 2.3. Differential Abundance Analysis and Visualization of LSIL/HSIL HPV Communities

The “Convert Abundance Table to Experiment” and “Proportion-based Statistical Analysis” runtimes on this dataset were 2 s and < 1 s, respectively (Table 1). The output generated a table listing the differential abundance and weighted proportions difference of each HPV type between LSIL and HSIL groups concomitant with statistical analysis (Baggerley’s test-statistic, *p*-value; Bonferroni and FDR corrected *p*-values).

**Figure 4:**
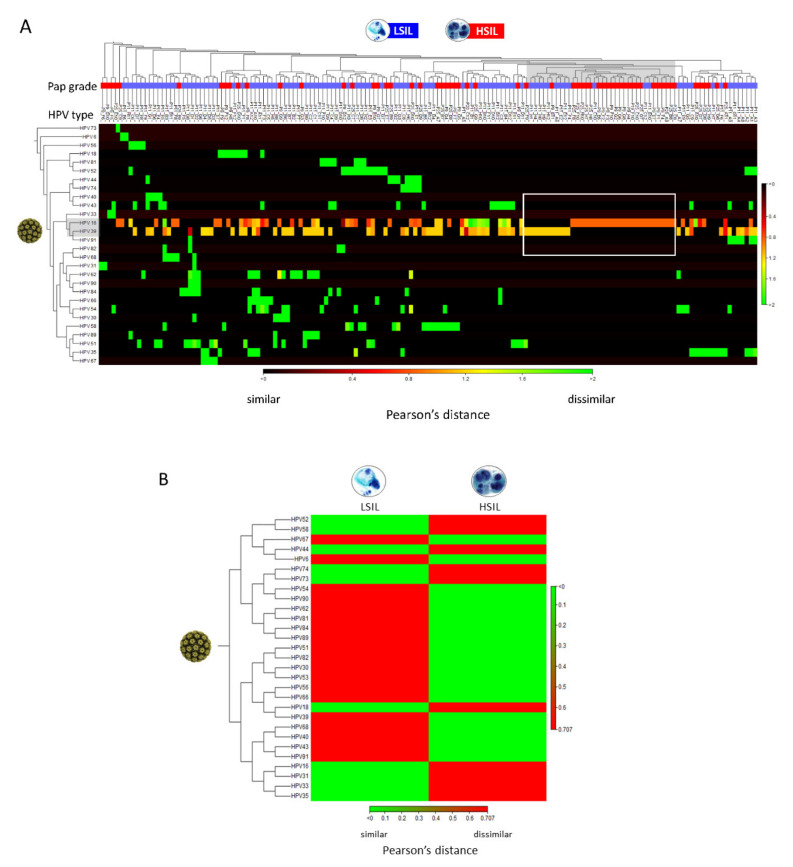
Clustered heat map of HPV abundance among LSIL/HSIL samples. (**A**) The heat map represents two-way hierarchal clustering of HPV over LSIL/HSIL samples (*n* = 155) and clustering of LSIL/HSIL samples over HPV. The dissimilarity measure i.e., Pearson’s distance (1- | Pearson correlation|) quantifies the dissimilarity in the variables of interest i.e., HPV type-specific abundance between individual samples. The agglomerative clustering method identifies the originating, most similar pair of clusters (gray shade). In this dataset, single (pure) infections of HPV-16 in HSIL and HPV-39 in LSIL of high abundance (rectangle) are the closest clusters. From this point, cluster divergence toward the left part of the heat map reveals increasingly, heterogeneous HPV infections predominantly in LSIL samples. The color scale shows Pearson’s distance between 0 (black) and 2 (green) indicating similar and dissimilar correlation coefficients, respectively. (**B**) Aggregated heat map of LSIL (*n* = 95) and HSIL (*n* = 60) samples reveal dissimilar, groupwise HPV profiles useful for data simplification and taxonomy development. The prevailing (abundant) genotypes for LSIL or HSIL are shown in red.

The “Create Heat Map for Abundance Table” runtime on this dataset was < 1 s. The resultant heat maps revealed hierarchal clustering of HPV genotypes on the 155 LSIL/HSIL samples. HSIL and LSIL samples with HPV genotypes, such as, HPV-16 and -39 with similarly high abundances (~100% of reads) are noted in the blue-red spectrum versus dissimilar abundance in the red spectrum (Figure 4A). The aggregated heat map of LSIL and HSIL samples revealed dissimilar HPV profiles with the prevailing genotypes shown in red (Figure 4B).

### 2.4. Read Mapping and Visualization of Mapped Tracks

**Figure 5:**
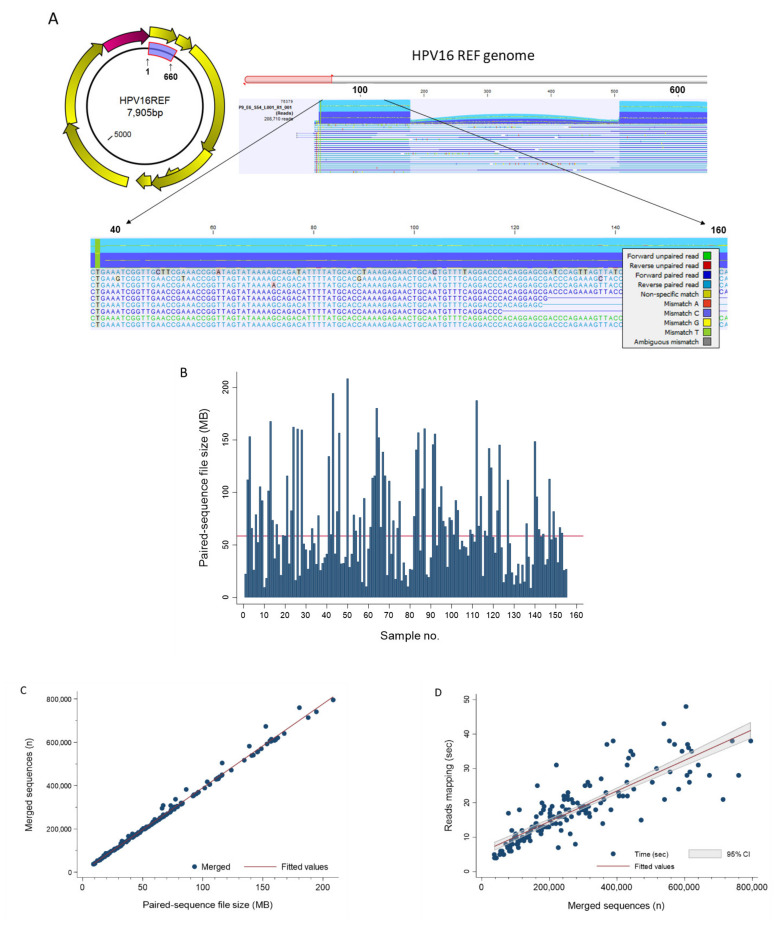
Read Mapping. (**A**) The HPV E6/E7 (660 bp) gene segment highlighted in blue on the circular prototypical HPV-16 genome (GenBank ID: K02718) is the target used for amplicon sequencing and genotyping. The Map Reads to Reference workflow output displays the reads mapped on to the linearized HPV reference genome. Zooming in from the whole genome window (top) allows viewing of the sequences down to the nucleotide level (bottom). The color-coding legend defines the corresponding read types and nucleotide mismatches. (**B**) NGS paired-sequence file size for each of the 155 study samples. The bar chart reveals the extent of file size variation between samples. The median (▬) was 58.5 MB (range, 8.9–208.5). (**C**) Scatterplot between NGS paired-sequence file size (MB) and merged sequences (*n*) for the 155 study samples showed near-perfect linear correlation (R^2^ = 0.9945). The regression line (merged sequences = 3415 + 3868 × file size) is shown as (▬). (**D**) Merged sequences (*n*) and reads mapping time (s) for the study cohort (*n* = 155) were highly correlated (R^2^ = 0.7233) as shown by the scatterplot and regression line (mapping time = 5.7 + 4.44 × 10^−5^ × merged sequences) (▬). The equations above may be used jointly or independently to estimate total mapping time based on file size or number of sequences.

The “Map Reads to Reference” workflow runtime on this dataset (*n* = 155) was 45:24 min with a mean of 18 s per sample (Table 1). The workflow generated two outputs: (1) mapping report and (2) reads track. Specifically, the mapping report summarized the total number of reads, mapped/unmapped reads, intact/broken paired reads, and matched/unmatched read length distribution per sample. A representative reads track shows 208,710 paired-reads of a sample mapped onto the linearized HPV-16 reference genome (Figure 5A). Mapping fortuitously revealed lower coverage (~14,000 to 32,000 reads) between nucleotide positions 178 and 507 versus the flanking regions (~73,000 reads) probably due to library preparation bias. Coverage bias or unevenness has been attributed to low-GC target regions, library preparation enzymes, library PCR amplification, cluster amplification, and sequencing [23,24,25]. Zooming in allowed visualization of the sequences down to the nucleotide level for comparison to the reference genome and detection of variants. Detailed analysis of mapping time revealed a high correlation between runtime and number of merged sequences in a sample. Furthermore, the number of merged sequences showed a linear correlation with input file size (MB). These correlative relationships are shown in Figure 5B–D. The fitted lines may be used independently or jointly to estimate mapping runtimes from sample file size or number of merged sequences.

## 3. Discussion

In this study, we developed and tested several workflows and tools for HPV virome profiling from deep sequenced clinical samples. By integrating our taxonomically customized HPV genome database within CLC MGM workflows, we were able to traverse disparate computational processes using one multi-functional software. Taxonomic classification and visualization of HPV metagenomes were accomplished efficiently and quickly to reveal differences between LSIL and HSIL viral communities. Alpha and Beta diversity analyses were processed in < 5 s to quantify the loss of α-diversity and gain of dominance by HPV-16 in HSIL over HPV-39 in LSIL samples. Similarly, differential abundance analysis and heat map visualization of LSIL/HSIL HPV communities were achieved within seconds to reveal dissimilar HPV profiles. The “Map Reads to Reference” workflow consumed more time than “Taxonomic Profiling” due to inherent computational complexity. The processing time correlated linearly with the number of merged sequences within a sample. The resulting mapped tracks with zoomable visualization provided easy inspection of mapped regions and detection of variants at the nucleotide-level. Finally, HPV genotyping results by NGS/taxonomic profiling was corroborated by concurrent Sanger/BLAST. In fact, NGS provided a much richer picture of the virome and evolutionary dynamics between disease states (i.e., LSIL to HSIL) than ever possible with conventional sequencing.

The strength of the methods developed herein is the integration of a taxonomized database with automated workflows for viral metagenomic analysis. The desired end state for the software user is a single, user-friendly, multi-functional tool, analogous to the “Swiss Army knife” [26]. We also strived to achieve the most efficient data processing pipeline by minimizing steps and time. Furthermore, testing the workflows on a portable notebook computer endorses software performance efficiency for benchtop or fieldwork, as well as, in austere clinical settings. In contradistinction, a recent publication used nine bioinformatics software for HPV de novo assembly, genotyping, phylogenetic analysis, and visualization (Bowtie2, SPAdes, VAPiD, MAFFT, RAxML, MEGA7, iTOL5.3, Dendroscope3, and R) [27,28,29,30,31,32,33,34,35,36]. In recent years, the list of superb bioinformatics software has lengthened considerably and is welcomed by the research community [18,37]. However, for the clinical virologist or physician-scientist, it is time-consuming and impractical to learn these sophisticated programs. Instead, a single, integrated, easy-to-use platform is preferred.

Another strength of this study is the corroboration of NGS/taxonomic profiling results by Sanger/BLAST. For the 9/155 (5.81%) discordant results, the quality of Sanger sequences was suboptimal in three samples, and primer or sequencing bias was the probable cause in six others. By convention, NGS results necessitate validation by Sanger sequencing as the reference standard. However, recently the necessity of Sanger validation has been called into question for human genome sequencing by several studies [38,39]. Arteche-Lopez et al., reported their validation study of 1109 NGS variants in 825 clinical exomes, the largest sample set to date using Illumina technology [38]. Only three discrepancies were found, and all false negative results arose from Sanger sequencing [38]. Taken together, high-quality NGS and analytical methods offer higher resolution and accuracy than Sanger/BLAST and only selective validation may be necessary. We acknowledge that our study has limitations that is, other cytological categories with potentially different HPV virome profiles were not included for comparison. To bridge this gap, we intend to analyze our ongoing large-scale study (>3000 samples) with the same workflows and streamline comparative analysis of three or more cytological categories.

## 4. Materials and Methods

### 4.1. Subjects, Samples, and Deep Sequencing

A subset of HPV-positive LSIL (*n* = 95) and HSIL (*n* = 60) cervical cytology samples were randomly selected from a larger (3000+ samples) Congressionally Directed Medical Research Programs (CDMRP) grant-funded project to test our workflow-based methods described herein. The residual liquid-based cervical cytology samples were procured consecutively from the Department of Pathology after completion of cytological diagnosis. Demographic and cytological data were abstracted from the electronic health record (AHLTA) as metadata for association with taxonomic profiling results.

DNA extraction, HPV DNA amplification and deep sequencing were performed as described previously with a few modifications below [14,40]. Cellular DNA extraction was performed after off-board cellular lysis using the QIAsymphony DSP DNA Midi Kit (96) in a QIAsymphony robotic workstation (Qiagen, Germantown, MD, USA). HPV DNA was amplified as previously published using consensus primers to target a 660 bp region of the E6/E7 gene (nucleotide positions 28 to 658 on the HPV-16 genome) for genotyping [14,40]. The PCR products were purified using PureLink (ThermoFisher Scientific, Waltham, MA, USA) spin column-based method and sent to Lucigen (Middleton, WI, USA) for next-generation sequencing. The submitted E6/E7 amplicons were mechanically sheared to 300–500 bp prior to construction of DNA libraries using the NxSeq AmpFREE low DNA library kit (Lucigen) per protocol. The four primary steps in library construction were: (1) end repair, (2) a-tailing, (3) adaptor ligation, and (4) size selection. The libraries were normalized quantitatively for equimolar representation from each sample prior to pooling and sequencing. Paired-end bi-directional sequencing (2 × 150 bp) was performed on the MiSeq instrument (Illumina, San Diego, CA, USA) using the MiSeq Reagent Kit v2 (300-cycles) for bridge amplification.

The PCR products were concurrently subjected to dideoxy (Sanger) sequencing for validation of deep-sequenced results. Briefly, amplicons (~200 ng DNA/sample) were sequenced using primer GP-E6-3F at Eurofins Genomics (Louisville, KY, USA). The resulting sequences were BLAST aligned for HPV genotyping as described above.

### 4.2. Customized HPV Reference Databases for CLC Workflows

**Figure 6:**
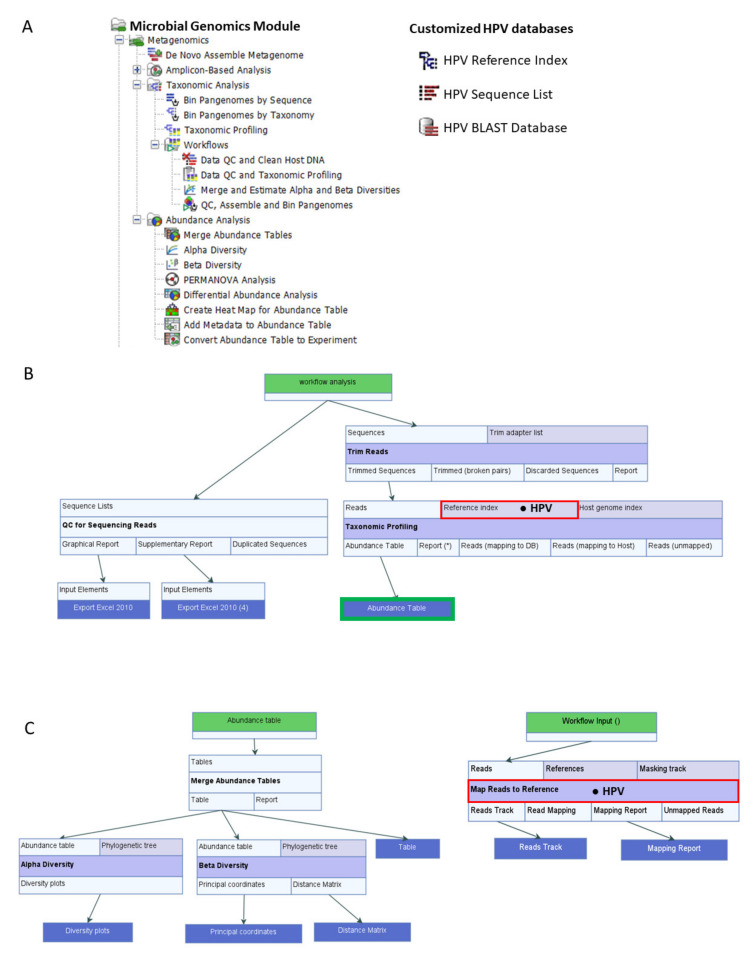
CLC Workflows, Tools, and Databases. (**A**) Microbial Genomics Module containing ready-to-use workflows and tools for abundance analysis (left). Customized HPV databases adapted from the PapillomaVirus Espiteme (PaVE) reference genomes (*n* = 219) for use in CLC (right). (**B**) Data QC and Taxonomic Profiling workflow incorporating the *HPV Reference Index* (● HPV) produces QC reports and HPV abundance tables from NGS reads of clinical samples. The Abundance Table output file (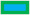) is utilized as the input file for downstream diversity analysis shown in (**C**). (**C**) Merge and Estimate Alpha and Beta Diversities workflow generates diversity plots and statistical results (left). Map Reads to Reference workflow incorporating the *HPV Sequence List* (● HPV) generates an alignment map of the reads on the reference HPV genomes (right).

HPV reference (*n* = 219) and variant genomes (*n* = 136) from the collection of NIAID PaVE (https://pave.niaid.nih.gov accessed on (11 December 2020) [21] were downloaded as GenBank files. The files were imported into CLC and customized for use as databases. Customization involved manual entry of author-defined, clinically relevant, common 7-level taxonomic nomenclature into each HPV genome file. Due to the lack of consensus in virus taxonomy and recent changes by the ICTV, our nomenclature was based on the PaVE classification with attributes from the Baltimore classification and the original ICTV 5-rank and current 15-rank taxonomic structures [19]. Specifically, we defined our 7-level taxonomic ranks as: Virus_nucleic acid type; Family; Genus; Species; Type; Lineage; and Sublineage. For HPV reference genomes, the taxonomic nomenclature was annotated to the 5th or “Type” level. For HPV variant genomes, the taxonomic nomenclature was annotated to the 7th or “Sublineage” level. For example, the taxonomy of HPV-16 reference genome was “Virus_dsDNA; Papillomaviridae; Alpha; Alpha 9; HPV16; blank; blank.” The taxonomy of an HPV-16 variant genome was defined to the sublineage level i.e., “Virus_dsDNA; Papillomaviridae; Alpha; Alpha 9; HPV16; A; A1.” Collectively, the taxonomized genome sequences were converted to 3 distinct CLC databases: (1) HPV Taxonomic Profiling Index, (2) HPV Sequence List, and (3) HPV BLAST database (Figure 6A). The database creation tools employed for this function were: Create Taxonomic Profiling Index, Sequence List, and Create BLAST Database. Finally, the three HPV reference databases were integrated and utilized within its respective workflows or tools: (1) Taxonomic Profiling, (2) Map Reads to Reference, and (3) multi-BLAST tool (Figure 6A).

### 4.3. Data Quality Control (QC) and Taxonomic Profiling Workflow

CLC Genomics Workbench 21.0.4 with CLC Microbial Genomics Module 21.0 (Redwood City, CA, USA) were installed on an HP notebook computer (specifications: Intel i7-7500U dual-core processor @ 2.70 GHz and 8 GB RAM) for use throughout this project. The MGM has various pre-built or “ready-to-use” workflows and tools as shown in Figure 6A. MGM offers two primary methods of metagenomic analysis (Amplicon-based OTU Clustering and Taxonomic Profiling). In amplicon-based analysis, the reads are clustered according to percentage of similarity (default 97%) into Operational Taxonomical Units (OTUs) which are then classified using a reference database [41]. In contrast, Taxonomic Profiling maps each input read to a reference database of complete genomes. Post-mapping read qualification and quantification are performed to generate the abundance table. The advantages of Taxonomic Profiling over OTU Clustering include fast and accurate identification and quantification of all qualifying reads in a metagenomic sample [42]. Furthermore, the resultant abundance table may be inputted directly into Abundance Analysis workflows or tools for visualization and statistical analysis [41]. 

The pre-built “Data QC and Taxonomic Profiling” workflow with data input/output, individual elements (processing steps) and flow directions (arrows) are presented in Figure 6B. The analysis consisted of four primary steps: Data import, Data QC, Taxonomic Profiling, and Visualization of Abundance Table. Each step is described in detail here. First, fastq files generated after sequencing were imported as paired-end (forward-reverse) and merged using the “Import Illumina” tool for placement into a new “input” folder created within the file structure. Second, the workflow was initiated by clicking “Run” to carry out these steps: (1) select input files, (2) preprocess reads with quality trimming based on quality scores with a limit cutoff 0.05, and the ambiguity number ≤ 2, and adapter trimming, (3) map and assign reads to the HPV reference index, and (4) quantify the abundance of each qualified HPV genotype to generate an abundance table for each sample.

The post-workflow finishing steps are described here. First, the resultant taxon tables were merged into one using the “Merge Abundance Tables” tool, cleaned by filtering out unmapped (genotype “Unknown”) sequences, and saved as a clean subtable. Second, the merged (filtered) taxon table was joined by clinical metadata (.xlsx format) with the “Add Metadata to Abundance Table” tool. Third, the two (merged, filtered, metadata-added) tables from LSIL (*n* = 95) and HSIL (*n* = 60) groups of samples were merged into one (*n* = 155) named “LSIL_HSIL abundance table” and aggregated by feature i.e., “Family” using the “Aggregate feature” option of the table (Supplementary Table S1). This option was applied to avoid lengthy feature names in the abundance table and graphs.

### 4.4. Estimate Alpha and Beta Diversities Workflow

Alpha diversity refers to species diversity within an ecosystem, whereas beta diversity denotes the change in species diversity between ecosystems. Several common alpha and beta diversity measures are available in MGM. The choice is based on the type of input data and biodiversity measurements sought by the researcher.

The pre-built “Merge and Estimate Alpha and Beta Diversities” workflow (Figure 6C) was used to analyze and compare HPV communities between groups, such as, cytological categories. The analysis consisted of three primary steps: Abundance table import and merge, Alpha Diversity and Beta Diversity analyses with visualization of diversity plots. The workflow was initiated by clicking “Run” to carry out these steps: (1) select cleaned LSIL and HSIL abundance tables with appended metadata, (2) merge abundance tables, (3) compute α-diversity of the HPV communities to measure within-sample variation, and (4) compute β-diversity of the HPV communities to measure between-sample variation. The choices for α-diversity measures included: Total number, Chao 1 bias-corrected, Chao 1, Simpson’s index, Shannon entropy, and Phylogenetic diversity. For this study, two α-diversity measures were computed, i.e., Simpson’s index [43]: SI = 1 $-\overset{n}{\underset{i=1}{\sum }}p_{i}^{2}$, and Shannon entropy [44]: H = $\overset{n}{\underset{1}{\sum }}p_{i}$ log_2_*p_i_*, where *n* was the number of HPV genotypes found in the sample, and *p_i_* was the proportion of reads that were identified as the *i*th HPV genotype. The workflow generated rarefaction curves, boxplots, and auto-calculated Mann-Whitney U statistical results comparing the alpha diversity indices of LSIL to HSIL groups. For β-diversity measures, the choices included: Bray-Curtis, Jaccard, Euclidean, and Phylogenetic diversity (UniFrac method with 5 variations). For this study, β-diversity or simply the change in HPV genotype composition (proportions) from the LSIL to HSIL group was measured by Bray-Curtis distances or compositional “dissimilarity” between samples [45]:

(1) $$Ɓ\;=\;\frac{\sum_{i=1}^{n}\left|x_{i}^{A}-x_{i}^{B}\right|}{\sum_{i=1}^{n}\left(x_{i}^{A}-x_{i}^{B}\right)}$$

where *n* is the number of operational taxonomic unit (OTU) *i* and $x_{i}^{A}\;\mathrm{and}\;x_{i}^{B}$ are the respective abundances of OTU *i* in samples *A* and *B.* The beta diversity workflow produced a Bray-Curtis distance matrix quantifying the dissimilarity of HPV genotype composition between samples. Also, Principal coordinate analysis (PCoA) was performed to determine and 3D-plot the correlative relationship between variables (HPV genotypes) in the LSIL or HSIL groups. The permutational multivariate analysis of variance (PERMANOVA) tool was used separately to test for statistical differences in the centroids and dispersion of the groups.

### 4.5. Differential Abundance Analysis Methods

For differential abundance analysis of HPV communities between LSIL and HSIL groups, the “Convert Abundance Table to Experiment” and “Proportion-based Statistical Analysis” tools were used in succession. First, the merged LSIL and HSIL abundance table with appended metadata was chosen as input for conversion. The metadata group named “PAP” with two categorical variables “LSIL” and “HSIL” was selected as the factor. The output produced a table labeled “experiment” which was entered into the “Proportion-based Statistical Analysis” tool as input. The weighted proportion test of Baggerly [46] was chosen for comparing proportions of a two-group experiment. Fundamentally, the test compares counts, such as, read counts in relation to total sum of counts in each sample. By comparing weighted proportions instead of counts, the data is corrected for sample size. For this study, changes (up- or downtrends) in HPV communities with disease progression from LSIL to HSIL was our primary interest. For visualization of differences in HPV communities, the “Create Heat Map for Abundance Table” tool was used to generate a heat map from the merged LSIL and HSIL abundance table. Heat map parameters were set with 1-Pearson correlation as the distance and Average linkage as the clustering method. Other distance measures (Euclidean and Manhattan distance) are available for calculating the distance between two features or samples. Additionally, cluster linkage methods (Single and Complete linkage) are available for measuring the distance between two clusters of features or samples [41].

### 4.6. Map Reads to Reference Workflow

The “Map Reads to Reference” workflow (Figure 6C) maps each sequencing read against the reference HPV Sequence List. The mapping consisted of three primary steps: import paired-end reads, map reads to reference genomes, and create reads track for visualization. The workflow was initiated by clicking “Run” to carry out these steps: (1) select paired-end reads, (2) map reads to reference, and (3) create reads track as output.

## 5. Conclusions

CLC workflows with integrated, customized HPV reference genomes proved to be an ultra-fast and accurate method of HPV virome genotyping and profiling. By forging a path through the bioinformatics pipeline of a user-friendly software, the impasses to HPV discovery and beyond have been overcome.

## Figures and Tables

**Table 1:** Software performance efficiency.

Workflow/Tool ^1^	Input File			Output	Runtime ^2^	
	*n*	Type	Size	File/Report/Table	Total	Unit
Import sequencing files	310	.fastq ^3^	4.4 GB	Merged paired-end reads in .clc format ^4^	00:03:20	<00:00:01
Data QC & Taxonomic profiling	155	.clc ^4^	10.6 GB	QC graphical reports Abundance table ^5^	00:36:23	00:00:14
Alpha and Beta diversities	1	.clc ^6^	4 KB	Diversity plots Distance matrix table ^5^	00:00:05	<00:00:01
Map reads to reference	155	.clc ^4^	10.6 GB	Mapping report Reads track	00:45:24	00:00:18
Differential abundance analysis	1	.clc ^6^	4 KB	Experiment table Statistical result table	00:00:03	<00:00:01
Convert abundance table to exp	1	.clc ^6^	4 KB	Experiment table Statistical result table	00:00:02	<00:00:01
Create heat map for abundance table	1	.clc ^6^	4 KB	Heat map chart	00:00:01	<00:00:01
BLAST ^7^	155	.phd ^8^	947 KB	BLAST table	00:00:12	<00:00:01

abun; abundance; BLAST, Basic Local Alignment Search Tool; .clc, CLC file format; dist., distance; E6/E7, HPV E6/E7 gene amplified by PCR; exp, experiment; HSIL, high-grade squamous intraepithelial lesion; QC, quality control; sec, second. ^1^ All workflows and tools were tested with the full dataset (*n* = 155) of samples. CLC pre-built workflows tested: (1) Data QC & Taxonomic profiling with integrated HPV reference genome database, (2) Alpha and Beta diversities, and (3) Map reads to reference. CLC microbial genomics tools tested: (a) Differential abundance analysis, (b) Convert abundance table to experiment, (c) Create heat map for abundance table, and (d) BLAST with HPV BLAST database. ^2^ Time notation (hh:mm:ss) represents the number of complete hours (hh), minutes (mm) and seconds (ss). Total time is the total runtime for 155 samples. Unit time is the mean runtime per sample. ^3^ Unmerged paired-end sequences in .fastq format. ^4^ Merged paired-end sequences in .clc format. ^5^ Table may be visualized as a chart with 1-click. ^6^ Merged LSIL and HSIL abundance table with appended metadata in .clc format. ^7^ BLAST (*blastn* program) united with the HPV reference and variant genome database was tested for genotyping Sanger sequences. Total runtime comprised of importing 155 sequences (1 s) + BLAST (11 s). ^8^ Sanger sequencing output in Phred (.phd) text files containing base calls and base-specific quality scores [22].

**Table 2:** HPV genotype concordance: Sanger seq/BLAST vs. Deep seq/Taxonomic profiling.

Agreement Statistic	LSIL	HSIL	LSIL/HSIL
Samples ^1^ (*n*)	95	60	155
Discordant ^2^ (*n*, %)	4 (4.21%)	5 (8.33%)	9 (5.81%)
Agreement ^3^ (*n*, %)	91 (95.79%)	55 (93.22%)	146 (94.81%)
Expected Agreement	12.95%	34.50%	15.11%
Kappa	0.9516	0.8965	0.9388
Std. Error	0.0345	0.0613	0.03
*p*-value	<0.0001	<0.0001	<0.0001

BLAST, Basic Local Alignment Search Tool; E6/E7, HPV E6/E7 gene amplified by PCR; HSIL, high-grade squamous intraepithelial lesion; HPV, human papillomavirus; L1, HPV L1 gene amplified by PCR; LSIL, low-grade squamous intraepithelial lesion; seq, sequencing. ^1^ Samples with HPV genotype(s) determined concurrently by Sanger sequencing/BLAST and deep sequencing/taxonomic profiling from HPV E6/E7 amplicons. The top ranking (most abundant and qualified) HPV genotype identified by taxonomic profiling was compared to the highest bit scoring (best) BLAST genotype. For non-sequenceable or interpretable HPV E6/E7 Sanger results, HPV L1 Sanger results were used alternatively. Detailed sequencing and genotyping results for individual samples (*n* = 155) are provided in Supplementary Table S2. ^2^ Samples with discordant HPV genotype results as determined by taxonomic profiling and BLAST. ^3^ HPV genotype agreement between the two sequencing/genotyping methods. For HPV variant lineage agreement, the result was nearly 100% agreement except for one sample (PC_2675) where the variant lineage of HPV type 68 could not be determined by deep sequencing. Distinct genotypes, variants, and sub-lineages, by definition, have > 10%, 1.0%, and 0.5% to 1.0% nucleotide sequence difference, respectively [21].

## Data Availability

The data presented in this study are openly available in the NCBI Sequence Read Archive (SRA). Title: HPV viromes in pap smears. SRA Accession Number: SRP323861; BioProject: PRJNA737277; BioSample Accession Numbers: SAMN19686938 to 19687092.

## Supplementary Materials

The following are available online at www.mdpi.com/article/10.3390/pathogens10081026/s1, Figure S1: Correlation between BLAST statistics, Table S1: LSIL and HSIL merged abundance table, Table S2: HPV E6/E7 Sanger and deep sequencing results, Table S3: BLAST results for HPV genotyping by Sanger sequencing. Reference [47] is cited in the supplementary materials.
