# Supplementary material for: HPV DeepSeq: An Ultra-Fast Method of NGS Data Analysis and Visualization Using Automated Workflows and a Customized Papillomavirus Database in CLC Genomics Workbench
Source: Pathogens. 2021 Aug 13;10(8):1026. doi: 10.3390/pathogens10081026 (PMC8398645; doi:10.3390/pathogens10081026)
Supplement: Supplementary file 1 [file pathogens-10-01026-s001.zip › pathogens-1290545 - Supplementary Materials (xml).pdf]

pathogens

Article

HPV DeepSeq: An Ultra-Fast Method of NGS Data Analysis and Visualization Using Automated Workflows and a Customized Papillomavirus Database in CLC Genomics Workbench

Jane Shen-Gunther [1,*], Qingqing Xia [2], Hong Cai [3,4] and Yufeng Wang [3,4,*]

1 Gynecologic Oncology & Clinical Investigation, Department of Clinical Investigation, Brooke Army Medical Center, Fort Sam Houston, TX 78234, USA
2 Department of Clinical Investigation, Brooke Army Medical Center, Fort Sam Houston, TX 78234, USA; qingqing.xia.civ@mail.mil
3 Department of Biology, University of Texas at San Antonio, San Antonio, TX 78249, USA; hong.cai@utsa.edu
4 South Texas Center for Emerging Infectious Diseases, University of Texas at San Antonio, San Antonio, TX 78249, USA
* Correspondence: jane.shengunther.mil@mail.mil (J.S.-G.); yufeng.wang@utsa.edu (Y.W.)

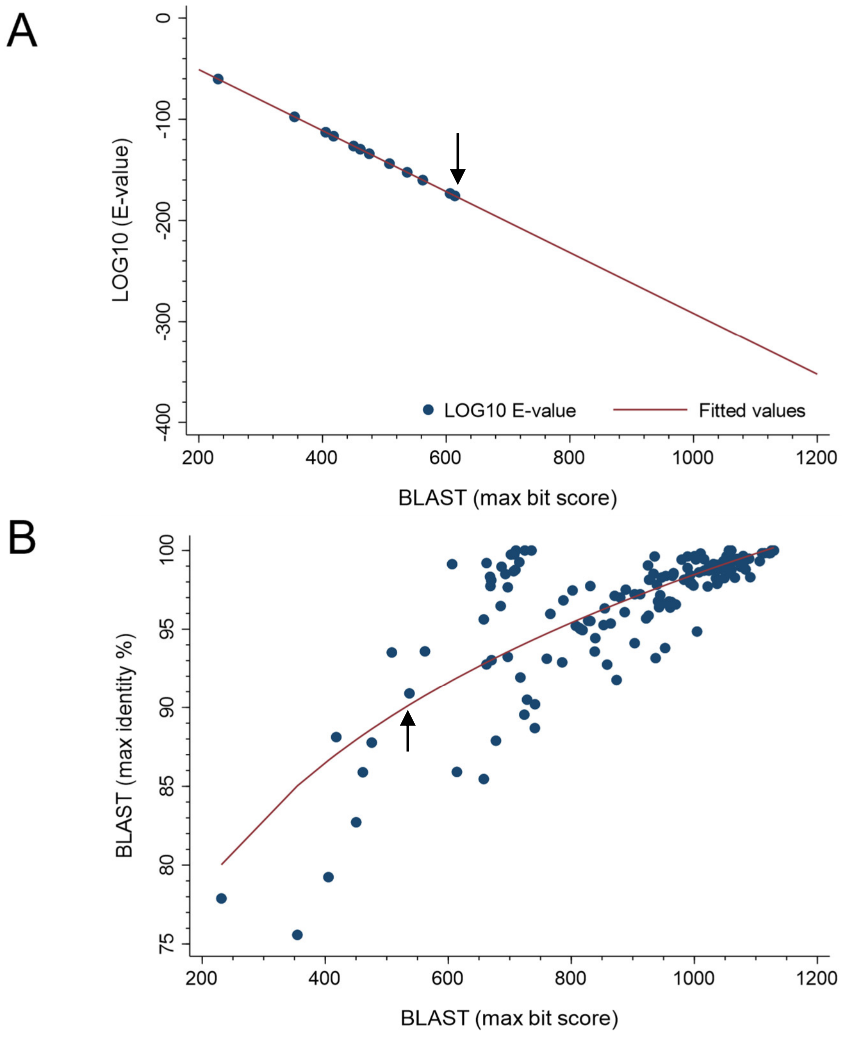

Figure S1. Correlation between BLAST statistics. (**A**) Scatterplot of BLAST maximum bit score and $\log_{10}$ E-value for the study samples (n = 154) showed a perfect linear negative-correlation (R^2 = 1.0). The regression line ($\log_{10}$ E-value = 9.41 + -0.301 * max bit score*)* shown as (—) may be employed for prediction of E-values based on bit scores. E-values < 2.13E-176 ($\log_{10}$ -176) equivalent to max bit scores > 614 (↓) were rounded and reported as 0 and interpreted as *identical* between the query and hit sequences in the BLAST report. Extrapolation of the fitted line allows for prediction of actual E-values based on bit scores (range 200-1,200) as shown. (**B**) Scatterplot of BLAST maximum bit score and maximum identity (%) for the study samples (n = 154) showed high curvilinear correlation (R^2 = 0.999). The least squares regression curve (max identity % = 37.02 * max bit score$^{0.1416}$) (—) based on the *power* equation model may be employed for prediction of max identity based on bit score. Bit scores < 531 equivalent to an E-value > 3.793E-151 ($\log_{10}$ -150) and max identity of < 90% (↑) may serve as a threshold for quality checks by manual verification of HPV genotype. Of note, one sample was excluded from the plot due to zero BLAST hits.

Table S2. HPV E6/E7 Sanger and deep sequencing results .

Sample Info [a]			Seq [b]	Deep Sequencing [c]														
				Reads Info [d]		HPV Genotypes and Proportional Composition [e]												
ID	PL	PAP	HPV Type	Total Merged Reads (*n*)	Total Mapped Reads (*n*)	HPV Type (n)	HPV#1	HPV#2	HPV#3	HPV#4	HPV#5	HPV#6	p#1	p#2	p#3	p#4	p#5	p#6
PC_2994	P11_A1	LSIL	39 A2	88,812	42,478	5	39 A	35	52	16	91	-	0.62	0.32	0.03	0.02	0.02	-
PC_3002	P11_A2	LSIL	35 A2	434,264	325,254	3	35 A1,2	39	52	-	-	-	0.96	0.03	0.01	-	-	-
PC_3010	P11_A3	LSIL	39 A2	592,196	451,184	4	39 A	52	91	35	-	-	0.71	0.26	0.02	0.01	-	-
PC_3018	P11_A4	LSIL	91	255,520	187,892	2	91	39	-	-	-	-	0.93	0.07	-	-	-	-
PC_3042	P11_A5	LSIL	39 A2	104,718	32,356	2	39 A	16	-	-	-	-	0.99	0.01	-	-	-	-
PC_3050	P11_A6	LSIL	39 A2	301,906	237,922	1	39 A	-	-	-	-	-	1.00		-	-	-	-
PC_3058	P11_A7	LSIL	39 A2	201,850	153,368	2	39 A	62	-	-	-	-	0.99	0.01	-	-	-	-
PC_3066	P11_A8	LSIL	39 A2	404,354	313,146	2	39 A	91	-	-	-	-	0.97	0.03	-	-	-	-
PC_3074	P11_A9	LSIL	91	353,110	261,928	4	91	39	43	54	-	-	0.75	0.12	0.10	0.02	-	-
PC_3082	P11_A10	LSIL	66 B1*	37,944	24,160	5	66 B1	39	35	16	62	-	0.53	0.36	0.06	0.04	0.01	-
PC_3003	P11_A11	LSIL	66 B1	71,374	26,432	6	66 B1	35	84	16	54	62	0.50	0.18	0.17	0.06	0.04	0.04
PC_3011	P11_A12	LSIL	35 A2	388,530	297,058	5	35 A1,2	62	66	16	54		0.74	0.14	0.05	0.04	0.02	-
PC_3019	P11_B1	LSIL	16 A1	640,246	495,266	2	16 A1	39	-	-	-	-	0.99	0.01	-	-	-	-
PC_3059	P11_B2	LSIL	39 A2	283,076	190,446	3	39 A	62	16	-	-	-	0.74	0.25	0.01	-	-	-
PC_3067	P11_B3	LSIL	16 A1	144,894	79,200	5	16 A1	68	39	43	82	-	0.90	0.04	0.03	0.01	0.01	-
PC_3075	P11_B4	LSIL	16 A1	265,916	216,072	3	16 A1	43	39	-	-	-	0.54	0.41	0.06	-	-	-
PC_3083	P11_B5	LSIL	58 C1	202,370	130,742	3	58 C1	43	39	-	-	-	0.72	0.26	0.01	-	-	-
PC_2996	P11_B6	LSIL	62	85,356	12,134	6	62	30	61	39	51	54	0.61	0.23	0.07	0.04	0.02	0.02
PC_3004	P11_B7	LSIL	30 A3	229,388	150,402	3	30 A	54	39	-	-	-	0.92	0.07	0.01	-	-	-
PC_3020	P11_B8	LSIL	30 A3	232,398	106,562	4	30 A2	39	51	89	-	-	0.54	0.42	0.03	0.01	-	-
PC_3028	P11_B9	LSIL	51	447,206	288,514	2	51 B1	89	-	-	-	-	0.81	0.19	-	-	-	-
PC_3036	P11_B10	LSIL	16 A1	124,796	59,988	2	16 A1	39	-	-	-	-	0.87	0.13	-	-	-	-
PC_3044	P11_B11	LSIL	39 A2	315,946	238,710	2	39 A	68	-	-	-	-	0.99	0.01	-	-	-	-
PC_3060	P11_B12	LSIL	39 A2	620,642	492,650	2	39 A	58	-	-	-	-	0.99	0.01	-	-	-	-
PC_3076	P11_C1	LSIL	39 A2	64,626	15,778	4	39 A	62	16	89	-	-	0.77	0.20	0.02	-	-	-
PC_3084	P11_C2	LSIL	39 A2	611,624	470,370	3	39 A	62	89	-	-	-	0.77	0.20	0.02	-	-	-
PC_3005	P11_C3	LSIL	**53 A1***	82,078	20,962	5	**16 A2**	39	43	84	66	-	0.47	0.36	0.14	0.02	0.01	-
PC_3013	P11_C4	LSIL	39 A2	608,524	459,216	4	39 A	43	84	81	-	-	0.69	0.27	0.03	0.01	-	-

PC_3029	P11_C5	LSIL	66 A1	201,624	73,564	3	66 A1	16	54	-	-	-	0.96	0.02	0.01	-	-	-
PC_3061	P11_C6	LSIL	16 C1	175,128	96,092	4	16 C1	52	89	56	-	-	0.96	0.02	0.01	0.01	-	-
PC_3069	P11_C7	LSIL	52 A1	106,440	50,024	5	52 A1	81	16	54	35	-	0.87	0.04	0.04	0.04	0.01	-
PC_3085	P11_C8	LSIL	54 A1	172,584	107,672	4	54 A1	35	74	39	-	-	0.96	0.02	0.01	0.01	-	-
PC_2998	P11_C9	LSIL	35 A2	253,604	168,400	4	35 A1,2	39	43	16	-	-	0.93	0.04	0.02	0.01	-	-
PC_3006	P11_C10	LSIL	16 A2	201,574	116,266	1	16 A1,2	-	-	-	-	-	1.00	-	-	-	-	-
PC_3014	P11_C11	LSIL	**53 A1***	132,216	13,684	4	**39 A**	16	62	89	-	-	0.66	0.16	0.16	0.02	-	-
PC_3030	P11_C12	LSIL	16 A1	300,230	207,512	1	16 A1	-	-	-	-	-	1.00	-	-	-	-	-
PC_3038	P11_D1	LSIL	91	102,316	29,464	6	91	51	82	90	84	39	0.75	0.13	0.06	0.04	0.01	0.01
PC_3046	P11_D2	LSIL	84	132,618	58,194	6	84	90	62	31	30	68	0.69	0.18	0.08	0.02	0.01	0.01
PC_3062	P11_D3	LSIL	51 A4	148,572	41,620	3	51 A4	39	67	-	-	-	0.96	0.03	0.01	-	-	-
PC_3086	P11_D4	LSIL	**35 A2**	160,108	101,614	3	**39 A**	67	35	-	-	-	0.56	0.29	0.15	-	-	-
PC_2999	P11_D5	LSIL	39 A2	517,018	351,454	3	39 A	67	35	-	-	-	0.62	0.33	0.05	-	-	-
PC_3007	P11_D6	LSIL	40	243,734	46,454	2	40	39	-	-	-	-	0.88	0.12	-	-	-	-
PC_3023	P11_D7	LSIL	39 A2	740,600	525,878	3	39 A	56	43	-	-	-	0.98	0.01	0.01	-	-	-
PC_3031	P11_D8	LSIL	58 A2	161,618	90,066	3	58 A2	52	81	-	-	-	0.72	0.25	0.03	-	-	-
PC_3039	P11_D9	LSIL	81	313,654	231,320	2	81	43	-	-	-	-	0.97	0.03	-	-	-	-
PC_3079	P11_D10	LSIL	43	603,936	461,732	1	43	-	-	-	-	-	1.00	-	-	-	-	-
PC_3000	P11_D11	LSIL	51 A3	133,102	16,160	3	51 A1	39	16	-	-	-	0.91	0.06	0.02	-	-	-
PC_3024	P11_D12	LSIL	39 A2	143,500	51,696	3	39 A	16	43	-	-	-	0.75	0.24	0.01	-	-	-
PC_3040	P11_E1	LSIL	**74**	154,566	13,726	4	**39 A**	44	74	16	-	-	0.55	0.23	0.22	0.01	-	-
PC_3048	P11_E2	LSIL	39 A2	795,470	614,336	1	39 A	-	-	-	-	-	1.00	-	-	-	-	-
PC_3064	P11_E3	LSIL	39 A2	112,554	27,900	4	39 A	51	52	16	-	-	0.95	0.02	0.02	0.01	-	-
PC_3072	P11_E4	LSIL	51 B2	176,666	27,338	3	51 B2	40	16	-	-	-	0.95	0.03	0.02	-	-	-
PC_3001	P11_E5	LSIL	40	305,320	196,398	3	40	51	43	-	-	-	0.64	0.35	0.01	-	-	-
PC_3009	P11_E6	LSIL	16 C1	243,784	189,034	2	16 C1	6	-	-	-	-	0.88	0.12	-	-	-	-
PC_3025	P11_E7	LSIL	67 A2	140,348	29,552	3	67 A2	16	51	-	-	-	0.75	0.22	0.03	-	-	-
PC_3049	P11_E8	LSIL	51 A	301,144	100,922	2	51 A4	56	-	-	-	-	0.97	0.03	-	-	-	-
PC_3057	P11_E9	LSIL	54 A1	60,022	9,190	3	54 A1	16	53	-	-	-	0.74	0.25	0.01	-	-	-
PC_3073	P11_E10	LSIL	16 A2	361,292	264,282	1	16 A1,2	-	-	-	-	-	1.00	-	-	-	-	-
PC_3081	P11_E11	LSIL	51 A3	41,084	10,078	3	51 A1	39	43	-	-	-	0.97	0.02	0.01	-	-	-
PC_2610	P11_E12	LSIL	39 A2	183,508	48624	1	39 A	-	-	-	-	-	1.00	-	-	-	-	-
PC_2642	P11_F1	LSIL	39 A2	265,374	138,400	3	39 A	16	18	-	-	-	0.93	0.06	0.01	-	-	-
PC_2690	P11_F3	LSIL	43	440,606	331,194	4	43	66	18	16	-	-	0.77	0.14	0.07	0.02	-	-

PC_2698	P11_F4	LSIL	39 A2	449,646	341,468	1	39 A	-	-	-	-	-	1.00	-	-	-	-	-
PC_2611	P11_F5	LSIL	84	759,680	146,418	1	84	-	-	-	-	-	1.00	-	-	-	-	-
PC_2651	P11_F6	LSIL	35 A1	673,276	76,172	2	35 A1	39	-	-	-	-	0.74	0.26	-	-	-	-
PC_2659	P11_F7	LSIL	62	308,150	36,790	1	62	-	-	-	-	-	1.00	-	-	-	-	-
PC_2675	P11_F8	LSIL	68 E1	582,066	57,072	2	68	16	-	-	-	-	0.98	0.02	-	-	-	-
PC_2683	P11_F9	LSIL	62	504,304	100,586	2	62	56	-	-	-	-	0.92	0.08	-	-	-	-
PC_2612	P11_F10	LSIL	54 A1	81,752	18,626	3	54 A1	39	81	-	-	-	0.90	0.09	0.01	-	-	-
PC_2620	P11_F11	LSIL	16 A1	429,260	337,404	2	16 A1	39	-	-	-	-	0.89	0.11	-	-	-	-
PC_2652	P11_F12	LSIL	18 A1	176,334	23,824	2	18 A1	39	-	-	-	-	0.93	0.07	-	-	-	-
PC_2676	P11_G1	LSIL	40	307,624	48,504	5	40	44	39	43	53	-	0.56	0.38	0.03	0.01	0.01	-
PC_2684	P11_G2	LSIL	58 A3	76,416	25,226	2	58 A2	44	-	-	-	-	0.60	0.40	-	-	-	-
PC_2692	P11_G3	LSIL	43	294,944	54,114	3	43	58	44	-	-	-	0.84	0.09	0.07	-	-	-
PC_2700	P11_G4	LSIL	39 A2	351,682	279,162	2	39 A	58	-	-	-	-	0.98	0.02	-	-	-	-
PC_2613	P11_G5	LSIL	54 A1	65,182	4,936	2	54 A1	39	-	-	-	-	0.99	0.01	-	-	-	-
PC_2621	P11_G6	LSIL	62	129,820	24,382	5	62	51	90	84	83	-	0.46	0.41	0.06	0.06	0.01	-
PC_2637	P11_G7	LSIL	52 A1	86,988	40,658	1	52 A1	-	-	-	-	-	1.00	-	-	-	-	-
PC_2661	P11_G8	LSIL	51 A4	43,236	17,496	2	51 A1	84	-	-	-	-	0.88	0.12	-	-	-	-
PC_2669	P11_G9	LSIL	81	108,428	64,810	2	81	66	-	-	-	-	0.59	0.41	-	-	-	-
PC_2677	P11_G10	LSIL	82 A2	106,552	33,860	2	82 A2	84	-	-	-	-	0.99	0.01	-	-	-	-
PC_2685	P11_G11	LSIL	91	336,892	45,024	1	91	-	-	-	-	-	1.00	-	-	-	-	-
PC_2693	P11_G12	LSIL	43	538,632	391,228	1	43	-	-	-	-	-	1.00	-	-	-	-	-
PC_2614	P11_H1	LSIL	39 A2	614,046	241,416	1	39 A	-	-	-	-	-	1.00	-	-	-	-	-
PC_2678	P11_H2	LSIL	52 A1	175,324	80,180	3	52 A1	58	81	-	-	-	0.89	0.10	0.01	-	-	-
PC_2686	P11_H3	LSIL	16 A2	417,468	194,648	3	16 A2	52	58	-	-	-	0.76	0.22	0.02	-	-	-
PC_2702	P11_H4	LSIL	39 A2	615,566	404,610	1	39 A	-	-	-	-	-	1.00	-	-	-	-	-
PC_2615	P11_H5	LSIL	66 B2	87,348	38,712	1	66 B2	-	-	-	-	-	1.00	-	-	-	-	-
PC_2655	P11_H6	LSIL	39 A2	87,386	21,386	4	39 A	84	44	74	-	-	0.70	0.23	0.05	0.03	-	-
PC_2663	P11_H7	LSIL	35 A2	166,216	61,464	1	35 A1,2	-	-	-	-	-	1.00	-	-	-	-	-
PC_2695	P11_H8	LSIL	39 A2	556,854	468,962	1	39 A	-	-	-	-	-	1.00	-	-	-	-	-
PC_2624	P11_H9	LSIL	39 A2	606,788	449,354	1	39 A	-	-	-	-	-	1.00	-	-	-	-	-
PC_2664	P11_H10	LSIL	16 A4	196,816	131,420	2	16 A1	39	-	-	-	-	0.68	0.32	-	-	-	-
PC_2680	P11_H11	LSIL	6 B1	382,046	62,782	1	6 B1	-	-	-	-	-	1.00	-	-	-	-	-
PC_2696	P11_H12	LSIL	39 A2	406,312	267,566	1	39 A	-	-	-	-	-	1.00	-	-	-	-	-
PC_3101	P9_E6	HSIL	16 A1	276,904	202,426	1	16 A1	-	-	-	-	-	1.00	-	-	-	-	-

PC_3109	P9_E7	HSIL	16 A1	257,900	182,236	1	16 A1	-	-	-	-	-	1.00	-	-	-	-	-
PC_3102	P9_E10	HSIL	51 A4	111,284	19,942	4	51 A4	39	52	16	-	-	0.91	0.05	0.03	0.01	-	-
PC_3110	P9_E11	HSIL	39 A2	290,156	202,112	1	39 A	-	-	-	-	-	1.00	-	-	-	-	-
PC_3142	P9_F1	HSIL	16 A1	287,112	121,304	1	16 A1	-	-	-	-	-	1.00	-	-	-	-	-
PC_3103	P9_F2	HSIL	16 A1	229,842	119,434	1	16 A1	-	-	-	-	-	1.00	-	-	-	-	-
PC_3111	P9_F3	HSIL	16 A1	357,738	139,506	1	16 A1	-	-	-	-	-	1.00	-	-	-	-	-
PC_3119	P9_F4	HSIL	16 A1	319,146	128,092	1	16 A1	-	-	-	-	-	1.00	-	-	-	-	-
PC_3127	P9_F5	HSIL	33 A1	178,144	67,688	1	33 A1,2	-	-	-	-	-	1.00	-	-	-	-	-
PC_3135	P9_F6	HSIL	31 B2	208,128	62,228	1	31 B1,2	-	-	-	-	-	1.00	-	-	-	-	-
PC_3143	P9_F7	HSIL	58 A2	191,080	29,722	1	58 A2	-	-	-	-	-	1.00	-	-	-	-	-
PC_3104	P9_F8	HSIL	39 A2	184,298	32,962	4	39 A	18	51	16	-	-	0.95	0.03	0.01	0.01	-	-
PC_3112	P9_F9	HSIL	**NA**	161,424	9,116	3	**35 A1,2**	16	18	-	-	-	0.94	0.04	0.02	-	-	-
PC_3120	P9_F10	HSIL	16 A1	251,164	136,528	1	16 A1	-	-	-	-	-	1.00	-	-	-	-	-
PC_3144	P9_F12	HSIL	18 A1	234,778	59,404	2	18 A1	58	-	-	-	-	0.84	0.16	-	-	-	-
PC_3121	P9_G1	HSIL	51 A2	209,376	93,372	1	51 A1	-	-	-	-	-	1.00	-	-	-	-	-
PC_3129	P9_G2	HSIL	16 A2	713,672	545,054	1	16 A1,2	-	-	-	-	-	1.00	-	-	-	-	-
PC_3137	P9_G3	HSIL	16 A1	261,602	127,236	2	16 A1	62	-	-	-	-	0.81	0.19	-	-	-	-
PC_3145	P9_G4	HSIL	58 A2	370,106	269,016	2	58 A2	54	-	-	-	-	0.96	0.04	-	-	-	-
PC_3106	P9_G5	HSIL	58 A3	78,976	10,948	1	58 A2	-	-	-	-	-	1.00	-	-	-	-	-
PC_3114	P9_G6	HSIL	16 A2	242,740	147,226	1	16 A1,2	-	-	-	-	-	1.00	-	-	-	-	-
PC_3122	P9_G7	HSIL	52 A1	226,378	116,138	2	52 A1	66	-	-	-	-	0.99	0.01	-	-	-	-
PC_3146	P9_G8	HSIL	16 A2	540,442	423,124	1	16 A1,2	-	-	-	-	-	1.00	-	-	-	-	-
PC_3107	P9_G9	HSIL	16 A2	471,444	349,684	3	16 A1,2	30	62	-	-	-	0.93	0.06	0.01	-	-	-
PC_3123	P9_G10	HSIL	31 C3	220,708	146,006	1	31 C1,3	-	-	-	-	-	1.00	-	-	-	-	-
PC_3131	P9_G12	HSIL	**62**	164,512	13,514	4	**52 A1**	82	16	59	-	-	0.96	0.02	0.01	0.01	-	-
PC_3140	P9_H4	HSIL	16 A1	317,592	188,426	1	16 A1	-	-	-	-	-	1.00	-	-	-	-	-
PC_3148	P9_H5	HSIL	16 A2	554,998	406,296	2	16 A1,2	18	-	-	-	-	0.61	0.39	-	-	-	-
PC_3125	P19_F10	HSIL	16 C1	182,394	116,072	1	16 C1	-	-	-	-	-	1.00	-	-	-	-	-
PC_1278	P20_C12	HSIL	16 A4*	55,922	33,290	2	16 A1,2	39	-	-	-	-	0.99	0.01	-	-	-	-
PC_3149	P25_A7	HSIL	58 B2*	90,408	37,374	3	58 B2	39	16	-	-	-	0.73	0.22	0.05	-	-	-
PC_3175	P25_A9	HSIL	16 C1	429,950	341,420	1	16 C1	-	-	-	-	-	1.00	-	-	-	-	-
PC_3187	P25_A10	HSIL	16 A4*	198,666	140,648	2	16 A1	35	-	-	-	-	0.99	0.01	-	-	-	-
PC_3172	P25_A11	HSIL	**16 A4***	97,068	61,986	3	**35 A1,2**	39	16	-	-	-	0.98	0.02	0.01	-	-	-
PC_3150	P25_B7	HSIL	**61***	53,576	6,394	3	**39 A**	16	58	-	-	-	0.86	0.13	0.01	-	-	-

PC_3163	P25_B8	HSIL	16 A4	83,768	34,466	1	16 A1	-	-	-	-	-	1.00	-	-	-	-	-
PC_3178	P25_B9	HSIL	16 A1	122,278	92,284	2	16 A1	44	-	-	-	-	0.99	0.01	-	-	-	-
PC_3153	P25_C7	HSIL	58 A2	53,648	14,764	3	58 A2	52	16	-	-	-	0.82	0.09	0.09	-	-	-
PC_3164	P25_C8	HSIL	16 C1	119,132	94,270	1	16 C1	-	-	-	-	-	1.00	-	-	-	-	-
PC_3179	P25_C9	HSIL	73 A1*	59,262	35,728	2	73 A2	16	-	-	-	-	0.88	0.12	-	-	-	-
PC_3190	P25_C10	HSIL	16 A4	269,908	212,336	1	16 A1	-	-	-	-	-	1.00	-	-	-	-	-
PC_3154	P25_D7	HSIL	35 A2	155,558	71,634	1	35 A1,2	-	-	-	-	-	1.00	-	-	-	-	-
PC_3167	P25_D8	HSIL	35 A2	37,264	16,864	2	36 A1,2	16	-	-	-	-	0.95	0.05	-	-	-	-
PC_3180	P25_D9	HSIL	16 A4*	122,100	86,816	3	16 A1,2	44	74	-	-	-	0.98	0.01	0.01	-	-	-
PC_3191	P25_D10	HSIL	16 A4*	570,044	348,482	5	16 A1,2	44	74	62	54	-	0.67	0.18	0.13	0.01	0.01	-
PC_3155	P25_E7	HSIL	39 A1*	367,438	308,654	2	39 A1	52	-	-	-	-	0.95	0.05	-	-	-	-
PC_3168	P25_E8	HSIL	16 A2	249,124	181,846	2	16 A2	58	-	-	-	-	0.67	0.33	-	-	-	-
PC_3181	P25_E9	HSIL	16 A4*	225,558	157,564	2	16 A1	68	-	-	-	-	0.99	0.01	-	-	-	-
PC_3192	P25_E10	HSIL	33 A2	238,546	123,548	2	33 A2	54	-	-	-	-	0.99	0.01	-	-	-	-
PC_3169	P25_F8	HSIL	16 A1	119,918	77,584	1	16 A1	-	-	-	-	-	1.00	-	-	-	-	-
PC_3182	P25_F9	HSIL	39 A2	139,590	97,146	2	39 A	51	-	-	-	-	0.99	0.01	-	-	-	-
PC_3193	P25_F10	HSIL	16 A1	430,734	368,762	1	16 A1	-	-	-	-	-	1.00	-	-	-	-	-
PC_3157	P25_G7	HSIL	16 A1	211,808	165,168	1	16 A1	-	-	-	-	-	1.00	-	-	-	-	-
PC_3173	P25_G8	HSIL	35 A2	319,774	204,232	1	35 A1,2	-	-	-	-	-	1.00	-	-	-	-	-
PC_3184	P25_G9	HSIL	16 A1	219,202	138,658	2	16 A1	39	-	-	-	-	0.97	0.03	-	-	-	-
PC_3195	P25_G10	HSIL	18 A2	136,122	40,256	1	18 A3,4	-	-	-	-	-	1.00	-	-	-	-	-
PC_3158	P25_H7	HSIL	16 A2	254,954	204,866	1	16 A2	-	-	-	-	-	1.00	-	-	-	-	-
PC_3174	P25_H8	HSIL	16 A4*	235,520	184,872	1	16 A1	-	-	-	-	-	1.00	-	-	-	-	-
PC_3186	P25_H9	HSIL	**83***	100,474	68,930	1	**35 A1,2**	-	-	-	-	-	1.00	-	-	-	-	-
PC_3171	P25_H10	HSIL	16 A4*	104,174	68,234	1	16 A1	-	-	-	-	-	1.00	-	-	-	-	-

E6/E7, HPV E6/E7 gene amplified by PCR; HSIL, high-grade squamous intraepithelial lesion; HPV, human papillomavirus; Info, information; ID, sample identification; L1, HPV L1 gene amplified by PCR; LSIL, low-grade squamous intraepithelial lesion; PCR, polymerase chain reaction; PL, PCR plate number and 96-well plate position; NA, not available/sequenceable; p #n, proportional composition of HPV genotype ranked in descending order by abundance; PAP, Pap smear; Seq, Sanger (dideoxy) sequencing; HPV #n, HPV genotype(s) identified ranked in descending order by abundance. [a] Sample ID, plate number and well position of LSIL (n = 95) and HSIL (n = 60) cervical cytology samples. [b] HPV genotype determined by BLAST alignment of E6/E7 amplicon/Sanger-based sequences. HPV genotypes are in numerals; variant lineage and sublineage are in alphanumeric values, respectively. For non-sequenceable or interpretable E6/E7 results, HPV L1 sequencing results were used alternatively for genotype determination. These samples (n = 16) are indicated by a star (*). Samples (9/155 samples, 5.81%) with discordant HPV typing results by Sanger sequencing/BLAST and deep sequencing/taxonomic profiling are in bold. [c] Sequence data and HPV genotype composition determined by the Taxonomic Profiling workflow. [d] Total merged and mapped reads statistics generated by the Taxonomic Profiling workflow. [e] HPV genotype(s) determined by taxonomic profiling of amplicon/deep seq-based sequences. HPV genotypes are in numerals; variant lineage and sublineage are in alphanumeric values, respectively. Up to 6 predominant HPV type(s) per sample are tabulated. Only

types meeting qualification (confidence score ≥ 0.995) and constituting ≥ 1% of the HPV composition within the sample) are listed. The reported confidence score equates to 1 - *p*-value under the null hypothesis that the reads map at random positions in the database [41]. The corresponding proportional composition are tabulated under p #n. Discordant HPV typing results by Sanger and deep sequencing are highlighted in bold (*n* = 9).

Table S3. BLAST results for HPV genotyping by Sanger sequencing.

Sample Info [a]					BLAST Statistics [b]											
**ID (Query)**	**PAP**	**AMP**	**HSP (*n*)**	**Lowest E-val**	**Accession (E-val)**	**HPV Type [c]**	**Hit [d]**	**Total Score**	**Max Score**	**Min E-Val**	**Max Bit Score**	**Max ID**	**Max % ID**	**Max Pos**	**Max % Pos**
PC_2994	LSIL	E6/E7	16	0	HPV39,_A2REF_KC470239	39,_A2	Access seq	487	487	0	937.04	531	93.16	531	93.16
PC (3002	LSIL	E6/E7	3	0	HPV35,_A2REF_HQ537727	35,_A2	Access seq	540	540	0	1038.94	556	98.06	556	98.06
PC_3010	LSIL	E6/E7	17	0	HPV39,_A2REF_KC470239	39,_A2	Access seq	522	522	0	1004.33	553	94.85	553	94.85
PC_3018	LSIL	E6/E7	1	0	HPV91REF	91	Access seq	521	521	0	1002.41	526	99.43	526	99.43
PC_3042	LSIL	E6/E7	16	0	HPV39,_A2REF_KC470239	39,_A2	Access seq	409	409	0	787.07	428	96.83	428	96.83
PC_3050	LSIL	E6/E7	17	0	HPV39,_A2REF_KC470239	39,_A2	Access seq	585	585	0	1125.46	585	100	585	100
PC_3058	LSIL	E6/E7	17	0	HPV39,_A2REF_KC470239	39,_A2	Access seq	577	577	0	1110.08	578	99.83	578	99.83
PC_3066	LSIL	E6/E7	17	0	HPV39,_A2REF_KC470239	39,_A2	Access seq	584	584	0	1123.54	585	99.83	585	99.83
PC_3074	LSIL	E6/E7	8	0	HPV91REF	91	Access seq	528	528	0	1015.87	533	99.44	533	99.44
PC_3082	LSIL	L1	231	0	HPV66,_B1REF_EF177188	66,_B1	Access seq	733	733	0	662.22	370	99.2	370	99.2
PC_3003	LSIL	E6/E7	9	0	HPV66,_B1REF_EF177188	66,_B1	Access seq	430	430	0	827.44	470	95.53	470	95.53
PC_3011	LSIL	E6/E7	11	0	HPV35,_A2REF_HQ537727	35,_A2	Access seq	531	531	0	1021.63	554	97.71	554	97.71
PC_3019	LSIL	E6/E7	11	0	HPV16,_A1REF_EU_PPH16	16,_A1	Access seq	550	550	0	1058.17	560	99.29	560	99.29
PC_3059	LSIL	E6/E7	17	0	HPV39,_A2REF_KC470239	39,_A2	Access seq	557	557	0	1071.62	564	99.3	564	99.3
PC_3067	LSIL	E6/E7	11	0	HPV16,_A1REF_EU_PPH16	16,_A1	Access seq	514	514	0	988.95	518	99.62	518	99.62
PC_3075	LSIL	E6/E7	11	0	HPV16,_A1REF_EU_PPH16	16,_A1	Access seq	385	385	0	740.92	497	90.2	497	90.2
PC_3083	LSIL	E6/E7	9	0	HPV58,_C1REF_HQ537774	58,_C1	Access seq	544	544	0	1046.63	553	98.93	553	98.93
PC_2996	LSIL	E6/E7	1	1.586E-144	HPV62REF	62	Access seq	264	264	1.59E-144	508.28	303	93.52	303	93.52
PC_3004	LSIL	E6/E7	17	0	HPV30,_A3REF_KF436844	30,_A3	Access seq	545	545	0	1048.55	555	98.93	555	98.93

PC_3020	LSIL	E6/E7	5	2.1342E-176	HPV30,_A3REF_KF436844	30,_A3	Access seq	319	319	2.13E-176	614.02	439	85.91	439	85.91
PC_3028	LSIL	E6/E7	18	0	HPV51,_B1REF_KF436883	51	Access seq	557	557	0	1071.62	564	99.3	564	99.3
PC_3036	LSIL	E6/E7	11	0	HPV16,_A1REF_EU_PPH16	16,_A1	Access seq	549	549	0	1056.24	559	98.59	559	98.59
PC_3044	LSIL	E6/E7	17	0	HPV39,_A2REF_KC470239	39,_A2	Access seq	579	579	0	1113.92	586	99.83	586	99.83
PC_3060	LSIL	E6/E7	17	0	HPV39,_A2REF_KC470239	39,_A2	Access seq	583	583	0	1121.61	585	99.83	585	99.83
PC_3076	LSIL	E6/E7	7	2.274E-117	HPV39,_A2REF_KC470239	39,_A2	Access seq	217	217	2.27E-117	417.91	282	88.12	282	88.12
PC_3084	LSIL	E6/E7	17	0	HPV39,_A2REF_KC470239	39,_A2	Access seq	575	575	0	1106.23	585	99.32	585	99.32
PC_3005	LSIL	L1	267	5.427E-174	HPV53,_A1REF_X74482	**53,_A1**	Access seq	671	671	5.43E-174	606.32	340	99.13	340	99.13
PC_3013	LSIL	E6/E7	17	0	HPV39,_A2REF_KC470239	39,_A2	Access seq	567	567	0	1090.85	577	98.3	577	98.3
PC_3029	LSIL	E6/E7	9	0	HPV66,_A1REF_HPU31794	66,_A1	Access seq	479	479	0	921.65	510	95.68	510	95.68
PC_3061	LSIL	E6/E7	11	0	HPV16,_C1REF,_AF2_AF472509	16,_C1	Access seq	563	563	0	1083.16	571	98.79	571	98.79
PC_3069	LSIL	E6/E7	8	0	HPV52,_A1REF_X74481	52,_A1	Access seq	446	446	0	858.21	486	92.75	486	92.75
PC_3085	LSIL	E6/E7	4	0	HPV54,_A1REF_HPU37488	54,_A1	Access seq	525	525	0	1010.1	526	99.81	526	99.81
PC_2998	LSIL	E6/E7	3	0	HPV35,_A2REF_HQ537727	35,_A2	Access seq	539	539	0	1037.02	557	98.76	557	98.76
PC_3006	LSIL	E6/E7	11	0	HPV16,_A2REF_EU_AF536179	16,_A2	Access seq	538	538	0	1035.09	554	98.23	554	98.23
PC_3014	LSIL	L1	275	0	HPV53,_A1REF_X74482	**53,_A1**	Access seq	740	740	0	668.53	389	97.74	389	97.74
PC_3030	LSIL	E6/E7	11	0	HPV16,_A1REF_EU_PPH16	16,_A1	Access seq	561	561	0	1079.32	569	99.3	569	99.3
PC_3038	LSIL	E6/E7	1	0	HPV91REF	91	Access seq	362	362	0	696.7	414	93.24	414	93.24
PC_3046	LSIL	E6/E7	2	0	HPV84REF	84	Access seq	398	398	0	765.92	429	95.97	429	95.97
PC_3062	LSIL	E6/E7	18	0	HPV51,_A4REF_KF436875	51,_A4	Access seq	367	367	0	706.31	374	98.68	374	98.68
PC_3086	LSIL	E6/E7	3	0	HPV35,_A2REF_HQ537727	**35,_A2**	Access seq	481	481	0	925.5	509	95.86	509	95.86
PC_2999	LSIL	E6/E7	7	0	HPV39,_A2REF_KC470239	39,_A2	Access seq	495	495	0	952.42	545	93.8	545	93.8
PC_3007	LSIL	E6/E7	1	0	HPV40REF	40	Access seq	344	344	0	662.09	397	92.76	397	92.76
PC_3023	LSIL	E6/E7	17	0	HPV39,_A2REF_KC470239	39,_A2	Access seq	586	586	0	1127.38	586	100	586	100
PC_3031	LSIL	E6/E7	9	0	HPV58,_A2REF_HQ537752	58,_A2	Access seq	528	528	0	1015.87	539	98.72	539	98.72
PC_3039	LSIL	E6/E7	1	0	HPV81REF	81	Access seq	514	514	0	988.95	536	98.89	536	98.89
PC_3079	LSIL	E6/E7	2	0	HPV43REF	43	Access seq	511	511	0	983.18	529	98.14	529	98.14
PC_3000	LSIL	E6/E7	18	0	HPV51,_A3REF_KF436873	51,_A3	Access seq	348	348	0	669.78	363	98.11	363	98.11
PC_3024	LSIL	E6/E7	17	0	HPV39,_A2REF_KC470239	39,_A2	Access seq	554	554	0	1065.86	568	98.27	568	98.27
PC_3040	LSIL	E6/E7	2	8.2989E-161	HPV74REF	**74**	Access seq	292	292	8.3E-161	562.11	336	93.59	336	93.59

PC_3048	LSIL	E6/E7	17	0	HPV39,_A2REF_KC470239	39,_A2	Access seq	583	583	0	1121.61	584	99.83	584	99.83
PC_3064	LSIL	E6/E7	9	0	HPV39,_A2REF_KC470239	39,_A2	Access seq	436	436	0	838.98	475	94.43	475	94.43
PC_3072	LSIL	E6/E7	18	0	HPV51,_B2REF_KF436886	51,_B2	Access seq	356	356	0	685.16	383	96.47	383	96.47
PC_3001	LSIL	E6/E7	1	0	HPV40REF	40	Access seq	376	376	0	723.62	471	89.54	471	89.54
PC_3009	LSIL	E6/E7	11	0	HPV16,_C1REF,_AF2_AF472509	16,_C1	Access seq	556	556	0	1069.7	559	99.47	559	99.47
PC_3025	LSIL	E6/E7	13	9.9772E-135	HPV67,_A2REF_HQ537780	67,_A2	Access seq	247	247	9.98E-135	475.59	330	87.77	330	87.77
PC_3049	LSIL	E6/E7	18	0	HPV51,A4REF_KF436875	51,_A	Access seq	454	454	0	873.59	502	91.77	502	91.77
PC_3057	LSIL	E6/E7	4	0	HPV54,_A1REF_HPU37488	54,_A1	Access seq	347	347	0	667.86	354	98.33	354	98.33
PC_3073	LSIL	E6/E7	11	0	HPV16,_A2REF_EU_AF536179	16,_A2	Access seq	560	560	0	1077.39	570	99.13	570	99.13
PC_3081	LSIL	E6/E7	18	0	HPV51,_A3REF_KF436873	51,_A3	Access seq	408	408	0	785.14	444	92.89	444	92.89
PC_2610	LSIL	E6/E7	58	0	HPV39,_A2REF_KC470239	39,_A2	Access seq	1066	1066	0	962.48	562	96.73	562	96.73
PC_2642	LSIL	E6/E7	58	0	HPV39,_A2REF_KC470239	39,_A2	Access seq	1138	1138	0	1027.4	579	98.81	579	98.81
PC_2690	LSIL	E6/E7	58	0	HPV43REF	43	Access seq	974	974	0	879.53	519	97.01	519	97.01
PC_2698	LSIL	E6/E7	58	0	HPV39,_A2REF_KC470239	39,_A2	Access seq	1174	1174	0	1059.86	587	100	587	100
PC_2611	LSIL	E6/E7	47	0	HPV84REF	84	Access seq	894	894	0	807.39	497	95.21	497	95.21
PC_2651	LSIL	E6/E7	60	0	HPV35,_A1REF_X74477	35,_A1	Access seq	1134	1134	0	1023.8	578	98.97	578	98.97
PC_2659	LSIL	E6/E7	57	0	HPV62REF	62	Access seq	964	964	0	870.51	506	97.12	506	97.12
PC_2675	LSIL	E6/E7	59	0	HPV68,_E1REF_KC470277	68,_E1	Access seq	1154	1154	0	1041.83	587	98.82	587	98.82
PC_2683	LSIL	E6/E7	64	0	HPV62REF	62	Access seq	902	902	0	814.61	495	95.01	495	95.01
PC_2612	LSIL	E6/E7	54	0	HPV54,_A1REF_HPU37488	54,_A1	Access seq	1028	1028	0	928.22	531	98.15	531	98.15
PC_2620	LSIL	E6/E7	55	0	HPV16,_A1REF_EU_PPH16	16,_A1	Access seq	1102	1102	0	994.94	570	97.94	570	97.94
PC_2652	LSIL	E6/E7	50	0	HPV18,_A1REF_AY262282	18,_A1	Access seq	1160	1160	0	1047.24	586	99.32	586	99.32
PC_2676	LSIL	E6/E7	15	2.75205E-98	HPV40REF	40	Access seq	392	392	2.75E-98	354.75	393	75.58	393	75.58
PC_2684	LSIL	E6/E7	65	3.8427E-127	HPV58,_A3REF_HQ537758	58,_A3	Access seq	528	498	3.84E-127	450.32	359	82.72	359	82.72
PC_2692	LSIL	E6/E7	61	0	HPV43REF	43	Access seq	1026	1026	0	926.41	529	98.14	529	98.14
PC_2700	LSIL	E6/E7	58	0	HPV39,_A2REF_KC470239	39,_A2	Access seq	1174	1174	0	1059.86	587	100	587	100
PC_2613	LSIL	E6/E7	54	0	HPV54,_A1REF_HPU37488	54,_A1	Access seq	920	920	0	830.84	475	97.74	475	97.74
PC_2621	LSIL	E6/E7	76	1.6181E-113	HPV62REF	62	Access seq	448	448	1.62E-113	405.24	374	79.24	374	79.24
PC_2637	LSIL	E6/E7	51	0	HPV52,_A1REF_X74481	52,_A1	Access seq	1108	1108	0	1000.35	557	99.64	557	99.64
PC_2661	LSIL	E6/E7	54	0	HPV51,_A4REF_KF436875	51,_A4	Access seq	944	944	0	852.48	523	95.26	523	95.26

PC_2669	LSIL	E6/E7	60	0	HPV81REF	81	Access seq	806	806	0	728.04	476	90.49	476	90.49
PC_2677	LSIL	E6/E7	50	0	HPV82,_A2REF_KF436787	82,_A2	Access seq	1070	1070	0	966.09	546	98.56	546	98.56
PC_2685	LSIL	E6/E7	57	0	HPV91REF	91	Access seq	888	888	0	801.98	460	97.46	460	97.46
PC_2693	LSIL	E6/E7	61	0	HPV43REF	43	Access seq	1034	1034	0	933.63	531	98.52	531	98.52
PC_2614	LSIL	E6/E7	58	0	HPV39,_A2REF_KC470239	39,_A2	Access seq	1106	1106	0	998.55	570	97.77	570	97.77
PC_2678	LSIL	E6/E7	51	0	HPV52,_A1REF_X74481	52,_A1	Access seq	1010	1010	0	911.99	525	97.22	525	97.22
PC_2686	LSIL	E6/E7	55	0	HPV16,_A2REF_EU_AF536179	16,_A2	Access seq	1100	1100	0	993.14	569	98.44	569	98.44
PC_2702	LSIL	E6/E7	58	0	HPV39,_A2REF_KC470239	39,_A2	Access seq	1170	1170	0	1056.26	593	99.16	593	99.16
PC_2615	LSIL	E6/E7	50	0	HPV66,_B2REF_EF177187	66,_B2	Access seq	1046	1046	0	944.45	548	97.16	548	97.16
PC_2655	LSIL	E6/E7	65	0	HPV39,_A2REF_KC470239	39,_A2	Access seq	820	820	0	740.67	494	88.69	494	88.69
PC_2663	LSIL	E6/E7	60	0	HPV35,_A2REF_HQ537727	35,_A2	Access seq	1116	1116	0	1007.57	569	98.61	569	98.61
PC_2695	LSIL	E6/E7	58	0	HPV39,_A2REF_KC470239	39,_A2	Access seq	1170	1170	0	1056.26	585	100	585	100
PC_2624	LSIL	E6/E7	58	0	HPV39,_A2REF_KC470239	39,_A2	Access seq	1170	1170	0	1056.26	585	100	585	100
PC_2664	LSIL	E6/E7	52	0	HPV16,_A4REF_ASIA_EA_AF534061	16,_A4	Access seq	728	728	0	657.71	482	85.46	482	85.46
PC_2680	LSIL	E6/E7	51	0	HPV_6,_B1REF_FR751337	6,_B1	Access seq	984	984	0	888.54	510	97.51	510	97.51
PC_2696	LSIL	E6/E7	58	0	HPV39,_A2REF_KC470239	39,_A2	Access seq	1170	1170	0	1056.26	593	99.16	593	99.16
PC_3101	HSIL	E6/E7	11	0	HPV16,_A1REF_EU_PPH16	16,_A1	Access seq	562	562	0	1081.24	574	99.31	574	99.31
PC_3109	HSIL	E6/E7	11	0	HPV16,_A1REF_EU_PPH16	16,_A1	Access seq	537	537	0	1033.17	551	99.1	551	99.1
PC_3102	HSIL	E6/E7	18	0	HPV51,_A4REF_KF436875	51,_A4	Access seq	352	352	0	677.47	450	87.89	450	87.89
PC_3110	HSIL	E6/E7	17	0	HPV39,_A2REF_KC470239	39,_A2	Access seq	587	587	0	1129.31	587	100	587	100
PC_3142	HSIL	E6/E7	11	0	HPV16,_A1REF_EU_PPH16	16,_A1	Access seq	539	539	0	1037.02	546	99.09	546	99.09
PC_3103	HSIL	E6/E7	11	0	HPV16,_A1REF_EU_PPH16	16,_A1	Access seq	545	545	0	1048.55	557	98.24	557	98.24
PC_3111	HSIL	E6/E7	11	0	HPV16,_A1REF_EU_PPH16	16,_A1	Access seq	561	561	0	1079.32	565	99.65	565	99.65
PC_3119	HSIL	E6/E7	11	0	HPV16,_A1REF_EU_PPH16	16,_A1	Access seq	551	551	0	1060.09	560	98.77	560	98.77
PC_3127	HSIL	E6/E7	6	0	HPV33,_A1REF_PPH33CG	33,_A1	Access seq	504	504	0	969.72	535	96.57	535	96.57
PC_3135	HSIL	E6/E7	11	0	HPV31,_B2REF_HQ537680	31,_B2	Access seq	395	395	0	760.15	460	93.12	460	93.12
PC_3143	HSIL	E6/E7	9	0	HPV58,_A2REF_HQ537752	58,_A2	Access seq	362	362	0	696.7	376	97.66	376	97.66
PC_3104	HSIL	E6/E7	16	0	HPV39,_A2REF_KC470239	39,_A2	Access seq	906	906	0	818.21	488	94.94	488	94.94
PC_3112	HSIL	L1	0		Not Available	NA	Access seq	NA	NA	NA	NA	NA	NA	NA	NA
PC_3120	HSIL	E6/E7	11	0	HPV16,_A1REF_EU_PPH16	16,_A1	Access seq	532	532	0	1023.56	542	98.72	542	98.72
PC_3144	HSIL	E6/E7	16	0	HPV18,_A1REF_AY262282	18,_A1	Access seq	449	449	0	863.97	473	95.36	473	95.36
PC_3121	HSIL	E6/E7	18	0	HPV51,_A2REF_KF436870	51,_A2	Access seq	509	509	0	979.34	514	99.42	514	99.42
PC_3129	HSIL	E6/E7	11	0	HPV16,_A2REF_EU_AF536179	16,_A2	Access seq	563	563	0	1083.16	574	98.8	574	98.8

PC_3137	HSIL	E6/E7	11	0	HPV16,_A1REF_EU_PPH16	16,_A1	Access seq	539	539	0	1037.02	553	97.88	553	97.88
PC_3145	HSIL	E6/E7	9	0	HPV58,_A2REF_HQ537752	58,_A2	Access seq	547	547	0	1052.4	551	99.64	551	99.64
PC_3106	HSIL	E6/E7	9	0	HPV58,_A3REF_HQ537758	58,_A3	Access seq	900	900	0	812.8	502	95.08	502	95.08
PC_3114	HSIL	E6/E7	11	0	HPV16,_A2REF_EU_AF536179	16,_A2	Access seq	1130	1130	0	1020.19	574	98.8	574	98.8
PC_3122	HSIL	E6/E7	8	0	HPV52,_A1REF_X74481	52,_A1	Access seq	502	502	0	965.88	517	98.48	517	98.48
PC_3146	HSIL	E6/E7	11	0	HPV16,_A2REF_EU_AF536179	16,_A2	Access seq	559	559	0	1075.47	569	98.96	569	98.96
PC_3107	HSIL	E6/E7	11	0	HPV16,_A2REF_EU_AF536179	16,_A2	Access seq	1142	1142	0	1031.01	577	99.14	577	99.14
PC_3123	HSIL	E6/E7	11	0	HPV31,_C3REF_HQ537685	31,_C3	Access seq	550	550	0	1058.17	559	99.11	559	99.11
PC_3131	HSIL	E6/E7	1	0	HPV62REF	**62**	Access seq	492	492	0	946.65	515	98.28	515	98.28
PC_3140	HSIL	E6/E7	11	0	HPV16,_A1REF_EU_PPH16	16,_A1	Access seq	552	552	0	1062.01	563	99.47	563	99.47
PC_3148	HSIL	E6/E7	11	0	HPV16,_A2REF_EU_AF536179	16,_A2	Access seq	489	489	0	940.88	509	96.77	509	96.77
PC_3125	HSIL	E6/E7	11	0	HPV16,_C1REF,_AF2_AF472509	16,_C1	Access seq	566	566	0	1088.93	569	99.48	569	99.48
PC_1278	HSIL	L1	273	0	HPV16,_A4REF_ASIA_EA_AF534061	16,_A4	Access seq	814	814	0	735.26	407	100	407	100
PC_3149	HSIL	L1	289	0	HPV58,_B2REF_HQ537764	58,_B2	Access seq	767	767	0	692.88	394	98.5	394	98.5
PC_3175	HSIL	E6/E7	144	0	HPV16,_C1REF,_AF2_AF472509	16,_C1	Access seq	946	946	0	854.28	497	96.32	497	96.32
PC_3187	HSIL	L1	257	0	HPV16,_A4REF_ASIA_EA_AF534061	16,_A4	Access seq	786	786	0	710.01	393	100	393	100
PC_3172	HSIL	L1	234	6.74034E-61	HPV16,_A4REF_ASIA_EA_AF534061	**16,_A4**	Access seq	255	255	6.74E-61	231.22	236	77.89	236	77.89
PC_3150	HSIL	L1	304	2.7766E-130	HPV61REF	**61**	Access seq	688	510	2.78E-130	461.14	341	85.89	341	85.89
PC_3163	HSIL	E6/E7	116	0	HPV16,_A4REF_ASIA_EA_AF534061	16,_A4	Access seq	742	742	0	670.34	427	93.03	427	93.03
PC_3178	HSIL	E6/E7	169	0	HPV16,_A1REF_EU_PPH16	16,_A1	Access seq	1062	1062	0	958.88	567	96.76	567	96.76
PC_3153	HSIL	E6/E7	129	0	HPV58,_A2REF_HQ537752	58,_A2	Access seq	794	794	0	717.22	467	91.93	467	91.93
PC_3164	HSIL	E6/E7	178	0	HPV16,_C1REF,_AF2_AF472509	16,_C1	Access seq	1044	1044	0	942.64	560	96.39	560	96.39
PC_3179	HSIL	L1	269	0	HPV73,_A1REF_X94165	73,_A1	Access seq	728	728	0	657.71	393	95.62	393	95.62
PC_3190	HSIL	E6/E7	157	0	HPV16,_A4REF_ASIA_EA_AF534061	16,_A4	Access seq	920	920	0	830.84	490	95.52	490	95.52
PC_3154	HSIL	E6/E7	200	0	HPV35,_A2REF_HQ537727	35,_A2	Access seq	1000	1000	0	902.97	525	97.22	525	97.22
PC_3167	HSIL	E6/E7	198	0	HPV35,_A2REF_HQ537727	35,_A2	Access seq	1040	1040	0	939.04	538	97.82	538	97.82
PC_3180	HSIL	L1	265	0	HPV16,_A4REF_ASIA_EA_AF534061	16,_A4	Access seq	785	785	0	709.11	402	98.77	402	98.77

PC_3191	HSIL	L1	280	0	HPV16,_A4REF_ASIA_EA_AF534061	16,_A4	Access seq	777	777	0	701.89	390	99.74	390	99.74
PC_3155	HSIL	L1	387	4.437E-153	HPV39,_A1REF_PPHT39	39,_A1	Access seq	1028	594	4.44E-153	536.89	359	90.89	359	90.89
PC_3168	HSIL	E6/E7	159	0	HPV16,_A2REF_EU_AF536179	16,_A2	Access seq	928	928	0	838.05	525	93.58	525	93.58
PC_3181	HSIL	L1	247	0	HPV16,_A4REF_ASIA_EA_AF534061	16,_A4	Access seq	783	783	0	707.3	393	99.75	393	99.75
PC_3192	HSIL	E6/E7	177	0	HPV33,_A2REF_HQ537698	33,_A2	Access seq	1056	1056	0	953.47	545	98.38	545	98.38
PC_3169	HSIL	E6/E7	164	0	HPV16,_A1REF_EU_PPH16	16,_A1	Access seq	1036	1036	0	935.43	521	99.62	521	99.62
PC_3182	HSIL	E6/E7	207	0	HPV39,_A2REF_KC470239	39,_A2	Access seq	1016	982	0	886.74	515	96.08	515	96.08
PC_3193	HSIL	E6/E7	150	0	HPV16,_A1REF_EU_PPH16	16,_A1	Access seq	1064	1064	0	960.68	558	96.37	558	96.37
PC_3157	HSIL	E6/E7	141	0	HPV16,_A1REF_EU_PPH16	16,_A1	Access seq	1068	1068	0	964.28	549	98.39	549	98.39
PC_3173	HSIL	E6/E7	182	0	HPV35,_A2REF_HQ537727	35,_A2	Access seq	1100	1100	0	993.14	566	97.92	566	97.92
PC_3184	HSIL	E6/E7	167	0	HPV16,_A1REF_EU_PPH16	16,_A1	Access seq	1050	1050	0	948.05	545	96.63	545	96.63
PC_3195	HSIL	E6/E7	178	0	HPV18,_A2REF_EF202146	18,_A2	Access seq	1024	1024	0	924.61	520	99.05	520	99.05
PC_3158	HSIL	E6/E7	145	0	HPV16,_A2REF_EU_AF536179	16,_A2	Access seq	1000	1000	0	902.97	543	94.11	543	94.11
PC_3174	HSIL	L1	263	0	HPV16,_A4REF_ASIA_EA_AF534061	16,_A4	Access seq	802	802	0	724.44	401	100	401	100
PC_3186	HSIL	L1	275	0	HPV83REF	**83**	Access seq	760	760	0	686.57	386	98.97	386	98.97
PC_3171	HSIL	L1	270	0	HPV16,_A4REF_ASIA_EA_AF534061	16,_A4	Access seq	792	792	0	715.42	400	99.26	400	99.26

Access seq, Accession sequence name; E6/E7, HPV E6/E7 gene amplified by PCR; HSIL, high-grade squamous intraepithelial lesion; HPV, human papillomavirus; Info, information; ID, sample identification; L1, HPV L1 gene amplified by PCR; LSIL, low-grade squamous intraepithelial lesion; NA, not available/sequenceable; PAP, Pap smear; PCR, polymerase chain reaction; Query, sequence used for BLAST search; Seq, Sanger (dideoxy) sequencing. [a] Sample ID of LSIL (n = 95) and HSIL (n = 60) cervical cytology samples subjected to HPV E6/E7 or L1 gene amplification by PCR. The amplified nucleotide sequences i.e., query sequences were BLAST-aligned for HPV genotyping. [b] BLAST statistics nomenclature and definitions from left to right of table columns [47]. HSP (n), Number of high scoring pairs from the source database. Lowest E-val, Lowest Expect value. Accession (E-val), Accession name of the matched database sequence with the lowest E-value. HPV Type, HPV genotype (numeral), variant and sub-lineage (alphanumeric) of the Accession sequence. Hit, Name of the sequence found in the BLAST search with the max score. Total score, Total alignment score for all HSPs. Max score, Maximum (best) score or highest alignment score of all HSPs. Min E-value, Minimum (best) e-value of all HSPs. Max bit score, Maximum (best) bit score of all HSPs. Max id, Maximum number of identical residues in the query and Hit sequence. Max %id, Percentage of maximum identical residues in the query and Hit sequence. Max pos, Maximum number of similar but not necessarily identical residues in the query and Hit sequence. Max %pos, Percentage of maximum similar but not necessarily identical residues in the query and Hit sequence. [c] HPV genotype as determined by BLAST alignment of E6/E7 amplicon/Sanger-based sequences and assigned according to the best Hit result. HPV genotypes are in numerals; variant lineage and sublineage are in alphanumeric values, respectively. For non-sequenceable or interpretable E6/E7 results, HPV L1 sequencing results (n = 16) were used alternatively for genotype determination. Samples (9/155 samples, 5.81%) with discordant HPV typing results by Sanger sequencing/BLAST and deep sequencing/taxonomic profiling are in bold. [d] Hit result with the max bit score were identical to the Accession sequence.
